# Incorporation of expanded organic cations in dysprosium(III) borohydrides for achieving luminescent molecular nanomagnets

**DOI:** 10.1038/s41598-021-88446-7

**Published:** 2021-05-31

**Authors:** Wojciech Wegner, Jakub J. Zakrzewski, Mikolaj Zychowicz, Szymon Chorazy

**Affiliations:** 1 College of Inter-Faculty Individual Studies in Mathematics and Natural Sciences, University of Warsaw, Banacha 2c, 02-097 Warsaw, Poland; 2 Center of New Technologies, University of Warsaw, Banacha 2c, 02-097 Warsaw, Poland; 3 Faculty of Chemistry, Jagiellonian University, Gronostajowa 2, 30-387 Kraków, Poland

**Keywords:** Chemistry, Inorganic chemistry, Physical chemistry

## Abstract

Luminescent single-molecule magnets (SMMs) constitute a class of molecular materials offering optical insight into magnetic anisotropy, magnetic switching of emission, and magnetic luminescent thermometry. They are accessible using lanthanide(III) complexes with advanced organic ligands or metalloligands. We present a simple route to luminescent SMMs realized by the insertion of well-known organic cations, tetrabutylammonium and tetraphenylphosphonium, into dysprosium(III) borohydrides, the representatives of metal borohydrides investigated due to their hydrogen storage properties. We report two novel compounds, [n-Bu_4_N][Dy^III^(BH_4_)_4_] (**1**) and [Ph_4_P][Dy^III^(BH_4_)_4_] (**2**), involving Dy^III^ centers surrounded by four pseudo-tetrahedrally arranged BH_4_^–^ ions. While **2** has higher symmetry and adopts a tetragonal unit cell (*I*4_1_/a), **1** crystallizes in a less symmetric monoclinic unit cell (*P*2_1_/c). They exhibit yellow room-temperature photoluminescence related to the f–f electronic transitions. Moreover, they reveal Dy^III^-centered magnetic anisotropy generated by the distorted arrangement of four borohydride anions. It leads to field-induced slow magnetic relaxation, well-observed for the magnetically diluted samples, [n-Bu_4_N][Y^III^_0.9_Dy^III^_0.1_(BH_4_)_4_] (**1@Y**) and [Ph_4_P][Y^III^_0.9_Dy^III^_0.1_(BH_4_)_4_] (**2@Y**). **1@Y** exhibits an Orbach-type relaxation with an energy barrier of 26.4(5) K while only the onset of SMM features was found in **2@Y**. The more pronounced single-ion anisotropy of Dy^III^ complexes of **1** was confirmed by the results of the ab initio calculations performed for both **1**–**2** and the highly symmetrical inorganic Dy^III^ borohydrides, α/β-Dy(BH_4_)_3_, **3** and **4**. The magneto-luminescent character was achieved by the implementation of large organic cations that lower the symmetry of Dy^III^ centers inducing single-ion anisotropy and separate them in the crystal lattice enabling the emission property. These findings are supported by the comparison with **3** and **4**, crystalizing in cubic unit cells, which are not emissive and do not exhibit SMM behavior.

## Introduction

Single-molecule magnets (SMMs) form an extraordinary class of d- or f-block metal complexes exhibiting strong magnetic anisotropy which results in the slow relaxation of magnetization^1–4^. Below so-called blocking temperature (*T*_B_), they reveal a magnetic hysteresis loop of a strictly molecular origin which opens their application horizon for high-density memory devices^5,6^. SMMs are also considered promising candidates for exploration in quantum computing as well as molecular spintronics^7–9^. The current state of art indicates that the strongest magnetic anisotropy, thus, the best performance SMMs, is achievable by playing with lanthanide(III) complexes^10–13^. The 4f metal centers reveal pronounced single-ion anisotropy due to the combined contributions from strong spin–orbit coupling and the crystal field effect which is much weaker but critical from the viewpoint of SMM features^14^. The axial distribution of negatively charged ligands around such lanthanide ions as Dy^3+^^13^, Tb^3+^^2^, or Ho^3+^^15^, is the most effective route for the generation of high-performance SMMs showing the record *T*_B_ values up to 80 K^16^.

Lanthanide single-molecule magnets are attractive as they offer also distinct photoluminescent properties related to their f-f electronic transitions^17,18^. Emissive lanthanide-based molecular materials arouse a broad scientific interest due to their numerous applications in such fields as optical storage^19^, optical communication^20^, bioimaging^21^, chemical sensing^22^, or optical thermometry^23^. The conjunction of photoluminescence and molecular nanomagnetism is an efficient tool for better understanding of magnetic anisotropy by investigating the high-resolution emission spectra which represent the electronic structure of the ground manifold of lanthanide ions^24,25^. Moreover, the bifunctionality offered by luminescent SMMs has been recently recognized to be promising from the viewpoints of the switching of emission by a magnetic field^26^, as well as luminescent thermometry for electromagnetic SMM-based devices exhibiting the self-monitoring of temperature^27–29^.

There are several synthetic pathways which were employed for achieving luminescent single-molecule magnets^18^, mainly based on visible light-emissive Dy^III^ and Tb^III^ centers^30,31^, NIR-emissive Yb^III^ or Nd^III^ centers^32,33^, or realized by the application of organic ligands as an emission source^34^. The majority of synthetic strategies applied in the construction of emissive SMMs take advantage of expanded organic ligands which are responsible both for constraining the coordination geometry of 4f metal ion towards strong single-ion anisotropy as well as sensitizing its luminescence through ligand-to-metal energy transfer^18,35^. Alternatively, luminescent molecular nanomagnets can be obtained by the incorporation of lanthanide ions into coordination frameworks, including metal–organic frameworks^36,37^, based on organic linkers or d-block metalloligands^38–40^. The up-to-date reported routes toward emissive SMMs explore rather sophisticated lanthanide(III) moieties exploring complicated organic ligands or a multi-component approach demanding the second metal complex^18,39,40^. Therefore, there is an attractive perspective in searching for simpler organic or inorganic molecular systems involving lanthanide(III) centers that can serve as luminescent molecular nanomagnets. In this context, we decided to test lanthanide(III) borohydrides as a possible source of luminescent SMMs.

The history of metal borohydrides starts in 1939 with the synthesis of the first member of this family, Al(BH_4_)_3_^41^. Interest in such systems was accelerated by the Manhattan Project, during which e.g. volatile actinide borohydrides were studied^42,43^. These studies laid the foundations for borohydride chemistry, by i.a. synthesis of NaBH_4_ and KBH_4_^44^ or preparation of other metal borohydrides using metathetic reactions on alkali metal borohydrides^45^. Since then, metal borohydrides were broadly investigated, mainly as reductive agents in chemical synthesis^46^. In the last 2 decades, the chemistry of metal borohydrides flourished again, as new metal borohydride systems were found promising as chemical hydrogen storage materials for fuel-cell vehicles^47–54^. Synthesis of new systems was also stimulated due to their various other properties, as metal borohydrides can serve as e.g. solid-state electrolytes in Li^+^ batteries^55–58^, organic catalysts^59,60^, precursors of metal borides^61–65^, or even boron nitride^66^. Lately, borohydrides of rare earth metal (RE) ions have been reported. Among them, binary yttrium borohydride is well-known and crystallizes in the same α-/β-RE(BH_4_)_3_ forms as lanthanide borohydrides^67,68^. In general, homoleptic lanthanide borohydrides crystalize in three polymorphic forms: r-RE(BH_4_)_3_ for RE = La, Ce^69^, Pr^70^, with trigonal unit cell; α-RE(BH_4_)_3_ and β-RE(BH_4_)_3_ for RE = Ce^69,71^, Pr, Nd^70^, Sm^72^, Eu^61^, Gd^61,68^, Tb^61,72^, Dy^61,68^, Ho^73^, Er^72,74^, Tm^61^, Yb^75^, Lu^71^, with cubic unit cells. The homoleptic RE(BH_4_)_2_ (with RE^*2*+^) compounds are also known for RE = Eu, Sm^76^, Yb^77^. The interest was also devoted to mixed cation borohydrides, containing lanthanide ions accompanied with alkali metal ions or organic groups. Rare earth borohydrides can exhibit strong photoluminescent properties originating from f-f or d-f electronic transitions, as was exemplified by α-Tb(BH_4_)_3_^78^ and the perovskite-type RE^2+^ borohydrides, KYb(BH_4_)_3_ and CsEu(BH_4_)_3_ together with CsCa(BH_4_)_3_:Eu^2+^^79^.

While the luminescent property was recognized in this family, the generation of magnetic anisotropy by using lanthanide borohydrides is a more challenging task as they usually crystallize in space groups of high symmetry with a rather isotropic coordination environment around 4f metal center. Moreover, a non-negligible super-exchange coupling was lately reported for both α-RE(BH_4_)_3_ and β-RE(BH_4_)_3_, RE = Gd, Tb, Dy, Ho, Er, Tm, along with KHo(BH_4_)_4_, RbTm(BH_4_)_4_ and MYb(BH_4_)_4_, M = Li, Na, showing that [BH_4_]^–^ is capable of mediating both weakly ferro- and antiferromagnetic super-exchange^80^. All these features are well-known to cancel the SMM effect of a single ion origin^14^. To overcome these difficulties, we decided to employ expanded organic cations, tetrabutylammonium (n-Bu_4_N^+^) and tetraphenylphosphonium (Ph_4_P^+^) for the construction of organic lanthanide(III) borohydrides to decrease the overall crystal symmetry and distort the geometry of 4f metal complexes. These are expected to generate distinct single-ion anisotropy and better magnetic isolation within the crystal lattice^81–83^. We decided to explore Dy(III) complexes which are yellow-to-white emissive as a result of the characteristic f-f electronic transitions^25,84^, and found to be highly anisotropic in diverse coordination environments.

As a result, we report the synthesis, crystal structures, magnetic and photoluminescent properties of two novel organic dysprosium(III) borohydrides, [n-Bu_4_N][Dy^III^(BH_4_)_4_] (**1**) and [Ph_4_P][Dy^III^(BH_4_)_4_] (**2**), first known derivatives of Dy(BH_4_)_3_, together with their magnetically Y^III^-diluted analogs, [n-Bu_4_N][Y^III^_0.9_Dy^III^_0.1_(BH_4_)_4_] (**1@Y**) and [Ph_4_P][Y^III^_0.9_Dy^III^_0.1_(BH_4_)_4_] (**2@Y**). They serve as luminescent molecular nanomagnets combining room-temperature Dy^III^-centered luminescence with a field-induced slow magnetic relaxation effect related to the intrinsic single-ion anisotropy of Dy^III^ centers surrounded by four borohydride anions. For the comparison, we also prepared and tested simple inorganic dysprosium(III) borohydrides, α/β-Dy(BH_4_)_3_, which do not reveal any luminescent or SMM features.

## Methods

### Synthesis, sample handling protocol, and chemicals used

All reactions, together with the sample storage and handling, were performed under an argon atmosphere in gloveboxes, with O_2_ and H_2_O levels < 1 ppm. All used reagents were anhydrous, of a high purity, purchased from Sigma-Aldrich: DyCl_3_, YCl_3_ > 99.99%; LiBH_4_ > 95%; (C_6_H_5_)_4_PCl (Ph_4_PCl) > 98%; (CH_3_CH_2_CH_2_CH_2_)_4_N(BH_4_) (n-Bu_4_NBH_4_) > 98%. Anhydrous dichloromethane (DCM), purchased from Sigma-Aldrich, was distilled over P_2_O_5_ and placed inside glovebox with the addition of molecular sieves. Mechanochemical reactions, using high energy milling method, were carried out in a milling bowl made of the diamagnetic form of SiC. Reactions were performed in the 5 min milling cycles, using a vibrational mill from Testchem (23.3 Hz, 1400 rpm). The milling vessel was cooled between cycles using liquid nitrogen to keep room temperature during the reactions and to avoid overheating of the samples and related thermal decomposition of the products. The synthesis and properties of both polymorphs of simple dysprosium borohydrides, α-Dy(BH_4_)_3_ and β-Dy(BH_4_)_3_, were described by us before^61^. Their preparation was performed according to the following reaction schemes:

1 $${\text{DyCl}}_{3} + 3{\text{ LiBH}}_{4} \to \, \upalpha {\text{ - Dy}}\left( {{\text{BH}}_{4} } \right)_{3} + 3{\text{ LiCl}}$$

2 $${\text{DyCl}}_{3} + 12{\text{ LiBH}}_{4} \to\upbeta {\text{ - Dy}}\left( {{\text{BH}}_{4} } \right)_{3} + 3{\text{ LiCl}} + 9{\text{ LiBH}}_{4}$$

Their novel organic derivatives, as well as those containing Y (magnetically diluted samples), were synthesized according to the reaction schemes:

3 $$\left[ {{\text{Ph}}_{4} {\text{P}}} \right]{\text{Cl}} + {\text{DyCl}}_{3} + 4{\text{ LiBH}}_{4} \to \left[ {{\text{Ph}}_{4} {\text{P}}} \right]\left[ {{\text{Dy}}\left( {{\text{BH}}_{4} } \right)_{4} } \right] + 4{\text{ LiCl}}$$

4 $$\left[ {{\text{Ph}}_{4} {\text{P}}} \right]{\text{Cl}} + 0.1{\text{ DyCl}}_{3} + 0.9{\text{ YCl}}_{3} + 4{\text{ LiBH}}_{4} \to \left[ {{\text{Ph}}_{4} {\text{P}}} \right]\left[ {{\text{Y}}_{0.9} {\text{Dy}}_{0.1} \left( {{\text{BH}}_{4} } \right)_{4} } \right] + 4{\text{ LiCl}}$$

5 $$\left[ {{\text{n - Bu}}_{4} {\text{N}}} \right]{\text{BH}}_{4} + {\text{ DyCl}}_{3} + 3{\text{ LiBH}}_{4} \to \, \left[ {{\text{n - Bu}}_{4} {\text{N}}} \right]\left[ {{\text{Dy}}\left( {{\text{BH}}_{4} } \right)_{4} } \right] + 3{\text{ LiCl}}$$

6 $$\left[ {{\text{n - Bu}}_{4} {\text{N}}} \right]{\text{BH}}_{4} + 0.1{\text{ DyCl}}_{3} + 0.9{\text{ YCl}}_{3} + 3{\text{ LiBH}}_{4} \to \, \left[ {{\text{n - Bu}}_{4} {\text{N}}} \right]\left[ {{\text{Y}}_{0.9} {\text{Dy}}_{0.1} \left( {{\text{BH}}_{4} } \right)_{4} } \right] + 3{\text{ LiCl}}$$

For the Y-diluted samples, in order to obtain single-phase, mixed metal Y^III^/Dy^III^ compound, rather than a physical mixture of single-metal compounds, the samples were additionally recrystallized from DCM. After dissolving [Cat][Dy_0.1_Y_0.9_(BH_4_)_4_], [Cat] = n-Bu_4_N or Ph_4_P, in DCM, the impurities in the form of LiCl were removed by decantation. Then, DCM was evaporated overnight allowing the crystallization of [Cat][Y_0.9_Dy_0.1_(BH_4_)_4_] in the desired form. Synthetic details for all samples are summarized in Table 1.

**Table 1:** Chemical composition of described compounds, their synthesis protocols, and used substrates.

Sample symbol	Substrates (mmol)	Synthesis protocol	Crystalline products
1	1 [n-Bu_4_N]BH_4_, 1 DyCl_3_, 3 LiBH_4_	12 × 5 min milling (5)	[n-Bu_4_N][Dy(BH_4_)_4_], LiCl
1@Y	1 [n-Bu_4_N]BH_4_, 0.1 DyCl_3_, 0.9 YCl_3_, 3 LiBH_4_	12 × 5 min milling (6), recrystallization and decantation from DCM	[n-Bu_4_N][Y_0.9_Dy_0.1_(BH_4_)_4_]
2	1 [Ph_4_P]Cl, 1 DyCl_3_, 4 LiBH_4_	12 × 5 min milling (3)	[Ph_4_P][Dy(BH_4_)_4_], LiCl
2@Y	1 [Ph_4_P]Cl, 0.1 DyCl_3_, 0.9 YCl_3_, 4 LiBH_4_	12 × 5 min milling (4), recrystallization from DCM	[Ph_4_P][Y_0.9_Dy_0.1_(BH_4_)_4_]
3	1 DyCl_3_, 3 LiBH_4_	4 × 5 min milling (1)	α-Dy(BH_4_)_3_, LiCl
4	1 DyCl_3_, 12 LiBH_4_	12 × 5 min milling (2)	0.9 β-/0.1α-Dy(BH_4_)_3_, LiCl, LiBH_4_

### Powder X-Ray diffraction (PXRD) studies and Rietveld refinement

PXRD measurements were performed using Bruker D8 Discover and Empyrean series 2 diffractometers, both with parallel beams. The CuK_α1_ and CuK_α2_ radiation, with an intensity ratio of ca. 2:1, was used in both cases. Samples were sealed under the argon atmosphere inside quartz capillaries (diameters of 0.5 or 1 mm). Rietveld refinement was performed using the Jana2006 program^85^. For **1** and **2**, preliminary models of [Ph_4_P][Tm(BH_4_)_4_]^83^ and [n-Bu_4_N][Y(BH_4_)_4_]^81^, respectively, were used. Pseudo-Voight functions were used to describe peak shapes, and, if needed, correction by divergence or Berar-Baldinozzi function was applied to describe peak asymmetry. The background was described by 36 (**1**) or 30 (**2**) Legendre polynomials. During the refinement, the following set of restrains was applied: B–H distances and H–B–H angles were fixed at 1.15 Å, with standard uncertainty (s.u.) = 0.001–0.01, and 109.47°, with s.u. = 0.01, respectively. The Dy–H distances (for three hydrogens from BH_4_^–^ coordinating Dy^III^) were fixed to be equal with s.u. = 0.01. Restrains were also applied to keep organic cations geometry. For [Ph_4_P]^+^ in **2** and **2@Y**, C–H distances were fixed at 1 Å (s.u. = 0.01), C–C at 1.4 Å (s.u. = 0.1), C–P at 1.8 Å (s.u. = 0.1), C–C–C angles were fixed at 120° (s.u. = 0.1) and C–P–C at 109.47° (s.u. = 0.01), additionally, phenyl groups were kept in planes. The Atomic Displacement Parameters (ADPs) for hydrogen atoms were fixed as 1.2 ADPs of boron atoms and for all P–C and B-B pairs of atoms were fixed to be equal. For [n-Bu_4_N]^+^ in **1** and **1@Y**, C–H distances were fixed at 1 Å (s.u. = 0.001), N–C and C–C to 1.5 Å (s.u. = 0.01); N–C–C, C–C–C, C–C–H and C–N–C angles were fixed at 109.47° (s.u. = 1). ADPs for C atoms were fixed to be equal, and ADPs of H atoms were fixed as 1.2 ADPs of C atoms. In BH_4_^–^ groups for all samples, ADPs for B atoms were fixed to be equal, and ADPs of H atoms were fixed as 1.2 ADP of B atoms. Visualization of structures was done with a Vesta software^86^. **CCDC:** [n-Bu_4_N][RE(BH_4_)_4_], RE = Dy (2058616), Y_0.9_Dy_0.1_ (2058617), and [Ph_4_P][RE(BH_4_)_4_], RE = Dy (2058615), Y_0.9_Dy_0.1_ (2058677), contain the crystallographic data for this paper which can be obtained through http://www.ccdc.cam.ac.uk/conts/retrieving.html or from the Cambridge Crystallographic Data Centre.

### Physical properties measurements and computational details

Alternate-current (*ac*) and direct-current (*dc*) magnetic measurements were performed using a Quantum Design MPMS 3 SQUID magnetometer with an Evercool system. Samples during the measurements were placed in the hermetic FEP capsules under an Ar atmosphere. Excitation and emission spectra were recorded on the Edinburgh Instruments FS5 spectrofluorometer equipped with a Xe arc lamp as an excitation source and a Hamamatsu photomultiplier as a detector. Computational details regarding the ab initio calculations of CASSCF/RASSI/SINGLE_ANISO type (together with the related references) performed on the experimental crystal structures of **1**–**4** were discussed in the Supporting Information.

## Results and discussion

### Synthesis outcome and crystal structures

#### [n-Bu_4_N][Y_1-x_Dy_x_(BH_4_)_4_] (**1**, x = 1; **1@Y**, x = 0.1) and[Ph_4_P][Y_1-x_Dy_x_(BH_4_)_4_] (**2**, x = 1; **2@Y**, x = 0.1)

**Figure 1:**
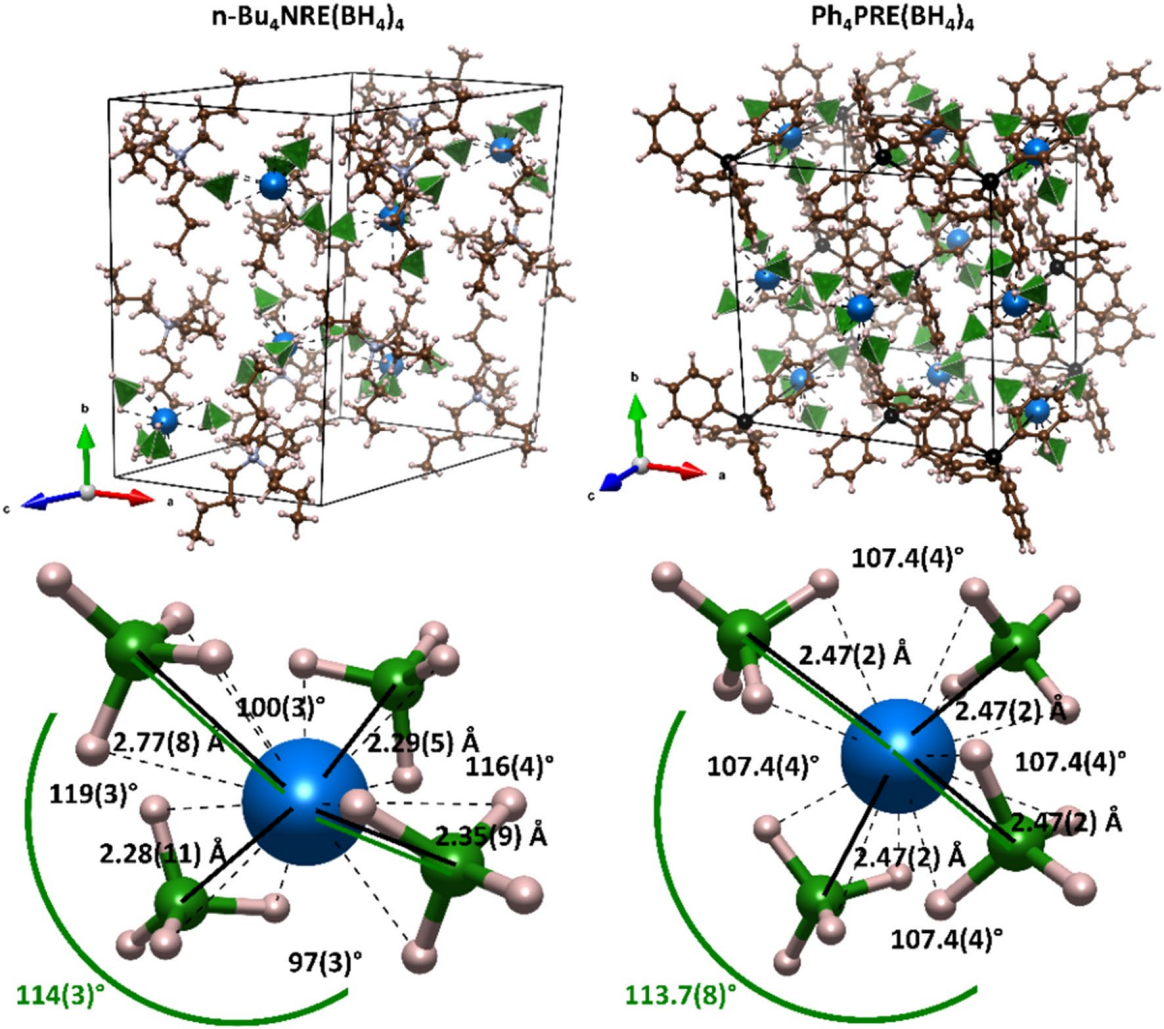
Crystal structures of **1** and **1@Y** (left) and **2** and **2@Y** (right): the unit cell contents (top) and the detailed insight into coordination environment of Dy centers (bottom). Colors: blue – RE^III^(Dy^III^); black – P; grey – N; brown – C; pink – H; green – B and BH_4_^–^ tetrahedron. Visualization presented for diluted samples.

Unit cells of [Cat][Dy(BH_4_)_4_], [Cat] = n-Bu_4_N^+^ (sample **1**) and Ph_4_P^+^ (sample **2**) are presented in Fig. 1, along with [Dy(BH_4_)_4_]^–^ geometries. They are constructed of alternating [Cat]^+^ and [Dy(BH_4_)_4_]^–^ ionic building blocks. The [RE(BH_4_)_4_]^–^ moiety is observed in almost all known derivatives of rare-earth (RE) borohydrides^87^. Each BH_4_^–^ group is serving as a tridentate ligand to RE^III^ (Dy^III^) metal center, leading to quasi 12-coordinated complexes. The crystal structure of **2** has higher symmetry and adopts a tetragonal unit cell (space group *I*4_1_/a), while **1** crystallizes in the monoclinic unit cell (*P*2_1_/c). In both structures, there is only one symmetrically independent cation (Ph_4_P^+^ or n-Bu_4_N^+^) and one independent anion [Dy(BH_4_)_4_]^–^. Additionally, in **2**, there is only one independent BH_4_^–^ group according to symmetry restrains, while in **1**, there are four of them, which leads to the deformed geometry of [Dy(BH_4_)_4_]^–^ in **1** (Fig. 1). In the supramolecular framework of **1**, the [Dy(BH_4_)_4_]^–^ unit is surrounded by nitrogen centers of four [n-But_4_N]^+^ cations forming a deformed trigonal pyramid, while each [n-Bu_4_N]^+^ is surrounded by four [Dy(BH_4_)_4_]^–^ anions in a distorted tetrahedron pattern. The crystal structure of **2** is of higher symmetry, and the closest Ph_4_P^+^ and [Dy(BH_4_)_4_]^–^ ions are arranged linearly along the *c* crystallographic axis. Considering the next closest counter-ions, Dy^3+^ ions are surrounded by four P-centers of Ph_4_P^+^ cations in a distorted tetrahedron pattern, and simultaneously the P-centers are surrounded by four Dy^3+^ ions. **1@Y** and **2@Y** have the same structures as analogous compounds built solely of Dy^III^ centers. In both as-milled samples (**1** and **2**), besides [Cat][Dy(BH_4_)_4_], only LiCl (metathesis by-product) is present but it doesn’t affect luminescence or magnetism. In **1@Y** and **2@Y**, LiCl was completely removed, as these samples need to be recrystallized to ensure the formation of [Cat][Y_0.9_Dy_0.1_(BH_4_)_4_] solid solution rather than the physical mixture of [Cat][Y(BH_4_)_4_] and [Cat][Dy(BH_4_)_4_]. Rietveld refinement plots for all compounds are placed in the Supporting Information, Figures S13–S16. It can be noted that intensities of PXRD patterns are noticeably higher for the diluted samples. This comes from the fact, that dysprosium samples were extremely sticky and could be placed only in capillaries with 1 mm diameter, which significantly reduces the XRD intensity. In diluted samples, some minor unidentified impurities could be seen in the PXRD patterns, and they were excluded from Rietveld refinement. It is worth mentioning that recent studies show that [n-Bu_4_N][RE^III^(BH_4_)_4_] compounds can crystallize in higher symmetric form, observed mainly for smaller RE^III^ (Tm, Yb, and Sc), named β-[n-Bu_4_N][RE^III^(BH_4_)_4_], while bigger ones (Tm, Ho, Y) tend to crystallize in lower symmetric form, α-[n-Bu_4_N][RE^III^(BH_4_)_4_]^81,82,88^. The latter is observed in the case of **1** and **1@Y**, further confirming the reported trend.

#### α-Dy(BH_4_)_3_ and β-Dy(BH_4_)_3_

Crystal structures and synthesis protocols for simple dysprosium borohydrides, α-Dy(BH_4_)_3_ (**3**) and β-Dy(BH_4_)_3_ (**4**), were described before^61,68^. Chemical compositions of these samples are presented in Table 1. In **3**, the only Dy-based phase is α-Dy(BH_4_)_3_, while **4** includes 90% of β- and 10% of α-Dy(BH_4_)_3_. Both LiCl (a by-product of the synthesis) and LiBH_4_ (used in excess in the reaction), present in these samples, are magnetically and luminescent inactive. Both α-Dy(BH_4_)_3_ and β-Dy(BH_4_)_3_ crystal structures consist of alternately arranged Dy^III^ and BH_4_^–^ units (Figure S17). They both adopt cubic unit cells, where six borohydride groups form octahedrons around each Dy^III^ center. This octahedron is regular in the case of β-Dy(BH_4_)_3_ and slightly distorted in the case of α-Dy(BH_4_)_3_ polymorph, which results in the lowering of local symmetry and slightly smaller Dy–Dy distances. Each borohydride group is shared between two dysprosium ions, coordinating each Dy^3+^ with two hydrogen atoms, leading to quasi 12-coordinated Dy centers.

For the comparison, we analyzed inorganic and organic derivatives of scandium borohydride, [Cat][Sc(BH_4_)_4_], [Cat] = Li^89^, Na^90^, K^91^, NH_4_^92^, Rb, Cs^93^, Me_4_N, n-Bu_4_N and Ph_4_P^82^, derivatives of yttrium borohydride, [Cat][Y(BH_4_)_4_], [Cat] = Li, Na^94^, K^95^, NH_4_^92^, Rb, Cs^96^, Me_4_N^95^, n-Bu_4_N^81^, and known derivatives of lanthanide borohydrides, [Cat][RE(BH_4_)_4_], such as RE = Er, [Cat] = Na^97^, K^98^; RE = Ho, [Cat] = K^73^; RE = Tm, [Cat] = Ph_4_P^83^; Rb^80^ or RE = Yb, [Cat] = Li^61^, Na, K^99^. Based on this analysis, [Cat] = n-Bu_4_N derivatives (including **1**) have the longest RE^III^–RE^III^ distances and the lowest unit cell symmetry. In the majority of cases, simple borohydrides have the shortest distances between RE^III^ centers. This trend is well illustrated in the obtained compounds (Table 2).

**Table 2:** Crystal data of **1** and **2**, and their Y-diluted analogs, compared with the reported **3** and **4**.

Compound	1	1@Y	2	2@Y	3	4
Composition	[n-Bu_4_N] [Dy(BH_4_)_4_]	[n-Bu_4_N] [Y_0.9_Dy_0.1_(BH_4_)_4_]	[Ph_4_P] [Dy(BH_4_)_4_]	[Ph_4_P] [Y_0.9_Dy_0.1_(BH_4_)_4_]	α-Dy(BH_4_)_3_^61,68^	β-Dy(BH_4_)_3_^61^
Space group	*P* 2_1_/c	*P* 2_1_/c	*I* 4_1_/a	*I* 4_1_/a	*P* a $\bar{3}$	*F* m $\bar{3}$ c
*a* [Å]	11.4240(10)	11.4464(7)	14.373(2)	14.3854(5)	10.8866(5)	11.05958(14)
*b* [Å]	20.531(2)	20.5665(17)	14.373(2)	14.3854(5)	10.8866(5)	11.05958(14)
*c* [Å]	15.2885(18)	15.3250(12)	13.560(3)	13.5715(5)	10.8866(5)	11.05958(14)
*V* [Å^3^]	2767.8(6)	2785.1(4)	2801.3(10)	2808.5(2)	1290.24(11)	1352.74(3)
*α* [°]	90	90	90	90	90	90
*β* [°]	129.478(8)	129.468(4)	90	90	90	90
*γ* [°]	90	90	90	90	90	90
*Z*	4	4	4	4	8	8
Dy–Dy [Å]	8.127(9)	–	7.9459(10)	–	5.4880(4)	5.52979(8)

### Magnetic properties: experiment and ab initio calculations

Direct-current (*dc*) magnetic properties for the powder samples of **1** and **2** are shown in Figure S1. Room-temperature values of magnetic susceptibility–temperature products, *χ*_M_*T*, are 14.5 and 14.3 cm^3^ mol^–1^ K for **1** and **2**, respectively, which are very close to the value of 14.2 cm^3^ mol^–1^ K, expected for the free-ion contribution from the single Dy^3+^ ion with its ^6^H_15/2_ ground multiplet. Upon cooling, the *χ*_M_*T* values gradually decrease, slowly in the 300–50 K range and more abruptly below this point. This behavior can be assigned to the Dy^III^ single-ion property, that is the thermal depopulation of the *m*_J_ levels within the ground manifold. However, non-negligible magnetic interactions between 4f metal centers, operating at low temperatures, cannot be excluded. They are rather weak due to the long Dy–Dy distances of 8.1 and 7.9 Å in **1** and **2**, respectively, but they cannot be fully neglected as the *χ*_M_*T* value drops quite abruptly in the lowest temperature regime reaching 6.9 cm^3^ mol^–1^ K at 1.8 K, which is a relatively small value when compared with typical magnetically isolated Dy^III^ complexes^32,100–102^. Magnetic interactions for **3** and **4** were described before^80^. They are of a weak ferromagnetic character with exchange constant, *J* of 0.018 and 0.033 cm^–1^, respectively (ferromagnetic character of the latter was confirmed by DFT calculations). **3** and **4** are built of Dy^III^ centers with intermetallic distances of ca. 5.5 Å enforced by bridging BH_4_^–^ ions, while in **1** and **2** [Dy(BH_4_)_4_]^–^ moieties are isolated by organic cations. The latter causes the increase of Dy–Dy distances by almost 50%, compared to inorganic salts, which reduces magnetic interactions. Magnetic-field-dependence of magnetization for **1** and **2** collected at *T* = 1.8 K reveals the featureless increase of the signal without full saturation even for the strong field of 7 T. This indicates the presence of non-zero magnetic anisotropy of Dy^III^ centers embedded in **1** and **2**. The highest achievable magnetization values of 5.9 and 6.1 μ_B_ for **1** and **2**, respectively, are within the typical range of 5–6 μ_B_ observable for magnetically anisotropic Dy^III^ complexes^32,100–102^. Magnetic hysteresis loops are not observed (Figure S2). The presence of significant magnetic anisotropy and/or low-lying excited states in **1** and **2** is also supported by the non-superposition of the reduced magnetization curves gathered at low temperatures below 5 K (Figure S3).

To examine the expected slow magnetic relaxation effect related to the presence of Dy^III^ moieties, alternate-current (*ac*) magnetic properties of **1** and **2** were investigated. Under zero *dc* field, even at the very low temperature of 1.8 K, **1** does not reveal any noticeable signal of the imaginary part of magnetic susceptibility, *χ*_M_”. The *χ*_M_” signal appears in the form of the high-frequency tail after applying a small *dc* field of 200 Oe (Figure S4). On the increasing *dc* field in the 200–1000 Oe range, the *χ*_M_” tail in the 10–1000 Hz improves but a further increase of the *dc* field leads to the disappearance of this relaxation. Such behavior may suggest the field-induced onset of some slow magnetic relaxation process related to the single-ion property of Dy^III^ appearing thanks to the canceling of the QTM effect at low fields and the further appearance of a direct process. To verify this hypothesis, we examined the *T*-dependence of the *ac* signal for the optimal *dc* field of 1 kOe. We found the gradual decreasing of the high-frequency *χ*_M_” tail from 1.8 to 3.0 K. No clear maxima were detected, thus, only the simplified approach based on the linear fragments of the ln(*χ*_M_”/*χ*_M_’) versus *T*^–1^ plots, following the method of E. Pardo et al., was used^103,104^. It gives the roughly estimated effective energy barriers related to the Arrhenius-type relaxation pathways. In **1** at 1 kOe, we found two linear parts of the ln(*χ*_M_”/*χ*_M_’) versus *T*^–1^ plots, operating at lower and higher temperatures, which indicate the presence of two effective energy barriers. However, they are very small, ca. 1.7 and 2.3 K, which is much below the limits for the typical Orbach relaxation of SMM systems. This suggests that the observed onset of magnetic relaxation is not of a pure single-ion origin, presumably affected by the non-negligible magnetic interactions between Dy centers. Such interpretation is supported by the appearance of the second, slower relaxation pathway at higher *dc* fields above 1.5 kOe as visualized by the new *χ*_M_” maxima in the 1–10 Hz range. We found that the related relaxation time, found from the *ac* signal following the generalized Debye model (Figure S5), is only weakly temperature-dependent as the increased temperature mainly leads to the weakening of the signal. The resulting *T*-dependence of relaxation time was also described by two linear fragments assignable to the Arrhenius-type processes but the very low energy barriers, ca. 2.5 and 7.2 K, accompanied by the unusually large τ_0_ values above 10^–3^ s, were determined (Table S1). This indicates that both relaxation processes detected in **1** should be ascribed mainly to dipolar interactions between insufficiently separated Dy centers.

**Figure 2:**
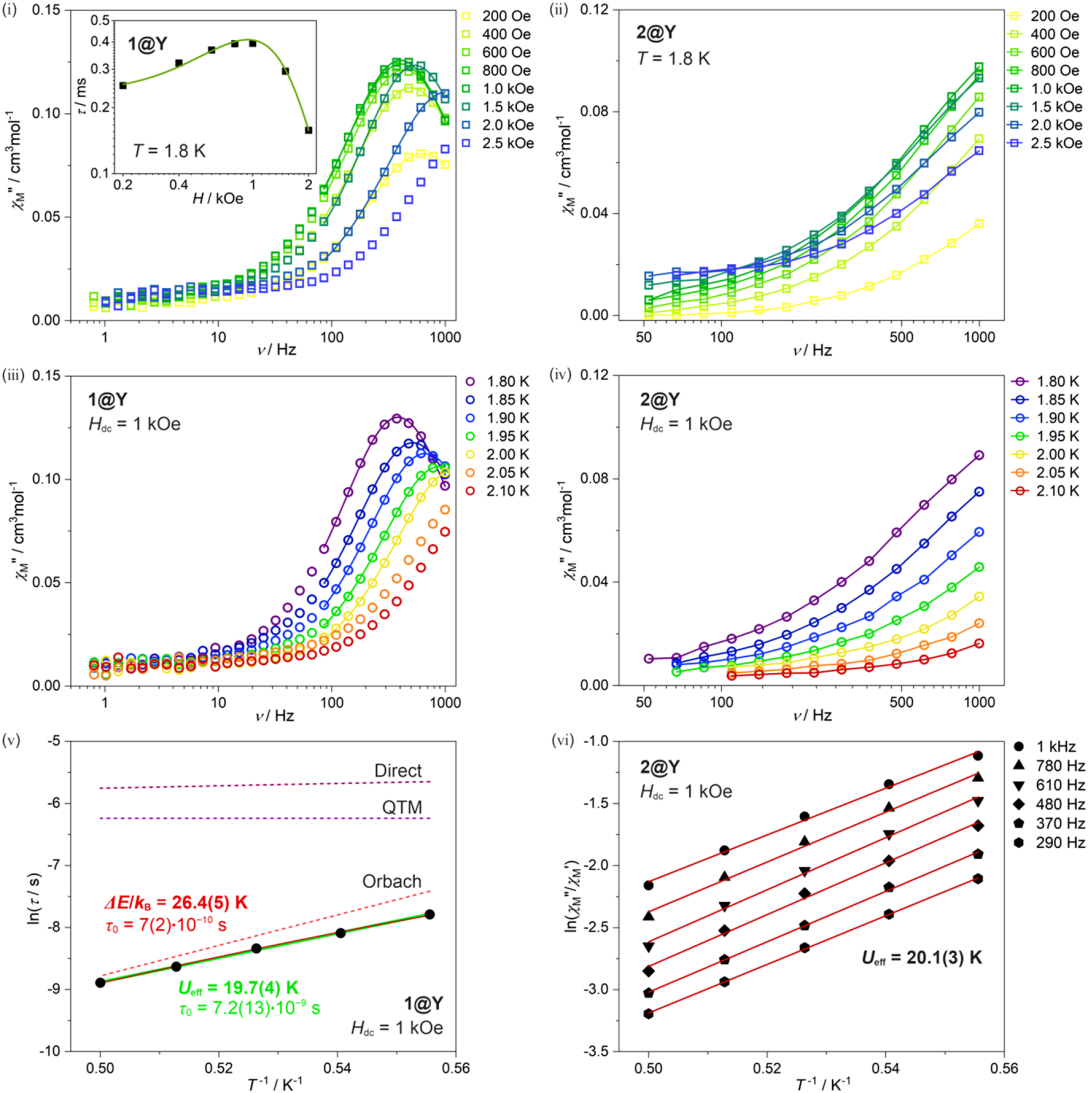
Dynamic (*ac*) magnetic properties of **1@Y** (left) and **2@Y** (right): *dc*-field-variable frequency dependences of the out-of-phase susceptibility, *χ*_M_” of **1@Y** (i) and **2@Y** (ii) gathered in the 200–2500 Oe range at 1.8 K, and the related field dependence of the relaxation time for **1@Y** (i), temperature-variable frequency dependences of the *χ*_M_” of **1@Y** (iii) and **2@Y** (iv) collected in the 1.8–2.1 K range at *H*_dc_ = 1 kOe, the related temperature dependence of the relaxation time for **1@Y** (v), and the ln(*χ*_M_”/*χ*_M_’) versus *T*^–1^ plots for the indicated frequencies of *ac* field (v). The empty colored points in the frequency dependences of the *χ*_M_” (i–iv) represent the experimental data. The solid lines in the respective curves of **1@Y** (i and iii) show the fitting according to the generalized Debye model while the analogous lines for **2@Y** (ii and iv) are only to guide the eye. The solid lines in the field- (i*,* green line) and temperature-dependences (v, dark red) of the relaxation time of **1@Y** represent the best-fit curve of the combined contributions from QTM, direct, and Orbach relaxation routes, while the black points show the experimental data. The light green line in (v), together with the green-colored text, represents the best-fit curves according to the Arrhenius law (the first term in the Eq. 7). The contributions from each type of relaxation were depicted in (v) as dotted lines. In the ln(*χ*_M_”/*χ*_M_’) versus *T*^–1^ plots of **2@Y** (vi), the black points show the experimental data while the solid red lines are the best-fit curves for a simplified approach elucidating the Arrhenius-type relaxation with the depicted effective energy barrier.

Therefore, we decided to prepare and investigate the magnetically diluted sample, **1@Y**, where Dy^III^ complexes are embedded in the diamagnetic Y^III^-based matrix (Figs. 2 and S6–S7). Even at the lowest temperature of 1.8 K, **1@Y** does not exhibit slow magnetic relaxation at zero *dc* field, however, the clear *χ*_M_” maxima appear after applying even the small *dc* field of 200 Oe (Fig. 2i). Starting from this field up to 2 kOe, the *χ*_M_”(ν), *χ*_M_’(ν), and Argand *χ*_M_”(*χ*_M_’) characteristics were analyzed using the Debye model for a single relaxation (Figure S6). The resulting *dc*-field-dependence of relaxation time reminds the typical behavior of the field-induced SMMs, that is the increasing *dc* field firstly slows down the magnetic relaxation in the low field range but it is further accelerated at higher fields. At the optimal *dc* field of 1 kOe, the *ac* magnetic curves were found to be strongly temperature-dependent indicating the Single-Molecule Magnet behavior (Figs. 2iii and S7). With increasing temperature, the *χ*_M_” maxima quickly move towards higher frequencies, crossing the available limit of 1000 Hz at ca. 2 K. The related *ac* plots were fitted using the Debye model. The resulting ln(*τ*) versus *T*^–1^ plot deviates from the linearity expected for the pure Orbach magnetic relaxation process of Arrhenius-type temperature dependence. This is not surprising as both QTM and direct process, as well as Raman relaxation effects, can operate for the case of a field-induced SMM. In general, the overall relaxation time (*τ*) being the function of *dc* field (*H*_dc_) and temperature (*T*) should be analyzed by considering four relaxation routes, using the Eq. (7):

7 $$\tau^{ - 1} = \tau_{0}^{ - 1} \exp \left( {\frac{{{\Delta }E}}{{k_{B} T}}} \right) + ATH^{4} + B_{Raman} T^{n} + \frac{{B_{1} }}{{1 + B_{2} H^{2} }}$$

Here, the first term represents the Orbach relaxation characterized by a thermal energy barrier, Δ*E*/*k*_B,_ and the relaxation attempt time, *τ*_0_. The second term is related to the direct process for a Kramers ion, depicted by the single parameter, *A*. The next term represents the Raman relaxation with two variable parameters, *B*_Raman,_ and the power *n*, adopting the values smaller or equal to 9 for the Kramers ions. The last term is related to the QTM effect which is only field-dependent and described by two parameters, *B*_1_ and *B*_2_^105^. To make an attempt on a reliable fitting following the Eq. (7), we applied the simultaneous fit of both field (Fig. 2i) and temperature dependences (Fig. 2v) of relaxation times. Even with this restriction, the number of accessible parameters is high (seven), thus, we considered various combinations of relaxation processes (Figs. 2v and S8, Tables S1–S2). The *τ* versus *H* dependence reveals the characteristic shape with the maximum around the optimal *dc* field of 1 kOe which can be interpreted in terms of the initial quenching of QTM and the further generation of a direct process. Therefore, we took into account two related contributions to the overall magnetic relaxation depicted by the parameters *A*, *B*_1,_ and *B*_2_, in which starting values were taken from the separate preliminary fit of the *τ* versus *H* plot. With this assumption, we tested the combination of QTM and direct processes together with Orbach or Raman relaxation processes. We found the successful fitting results by using three relaxation processes, QTM, direct, and Orbach (Figs. 2i, 2v). The best-fit parameters are Δ*E*/*k*_B_ = 24.6(5) K, *τ*_0_ = 7(2)·10^–10^ s, *A* = 1.58(3)·10^–10^ s^–1^ K^–1^Oe^–4^, *B*_1_ = 2619(136) s^–1^, and *B*_2_ = 4.1(6)·10^–6^ Oe^–2^. The parameters for Orbach relaxation, Δ*E*/*k*_B,_ and *τ*_0_, are within the ranges characteristic of lanthanide SMMs, however, of a rather weak magnetic anisotropy. The alternative fitting employing Raman relaxation, instead of Orbach process, along with QTM and direct relaxation routes, was excluded as it resulted in the unrealistically high power *n* of 12.9(3) for the Raman process, while the fixed power *n* of 9 leads to the poor quality of the fitting (Figure S8 and Table S2). As the observed contributions from QTM and direct processes to the overall magnetic relaxation (Fig. 2v) are weak, we also examined the fitting procedures without these two processes. In this context, we found that Raman relaxation cannot reliably describe the *T*-dependence of relaxation time due to the unrealistic parameters or poor fitting quality (Figure S8 and Table S2) while the sole Orbach relaxation relatively well represent the experimental data giving the slightly decreased energy barrier of 19.7(4) K accompanied by the pre-exponential factor of 7.2(13)·10^–9^ s (Figs. 2v and S8e). These last values correspond to the simple Arrhenius law (the first term in the Eq. 7), thus they give insight into the effective energy barrier (*U*_eff_) and the pre-exponential factor (*τ*_0_) which were found to be typical for moderate Dy(III) SMMs. Nevertheless, the above-presented analyses suggest that the borohydride Dy^III^ complexes of **1@Y** at the accessible temperature regime (1.8–2 K) exhibits three relaxation processes, QTM, direct, and the dominant Orbach. The determination of other possible relaxation, a Raman process, is not achievable due to the limited temperature range. Only the access to lower than the 1.8 K temperatures for *ac* measurements may elucidate this issue.

Qualitatively similar *ac* dynamic properties were detected for **2** and its magnetically diluted sample, **2@Y** (Figs. 2 and S9–S10). Similarly to **1**, compound **2** exhibits the field-induced *ac* magnetic signal. However, it appears only in the low-frequency region below 50 Hz as the signal tails without the clear maximum in the range of 0.5–1000 Hz (Figure S9). We followed the *T*-dependence of this magnetic relaxation for the optimal *dc* field (i.e. those of the strongest *χ*_M_” signal) of 1 kOe finding the gradual disappearance of the *χ*_M_” tails without any frequency shifts. Therefore, the observed relaxation process in **2** was assigned to the dipolar interactions between Dy^III^ centers. The lack of any *ac* signal in the higher frequency range, as was detected in **1**, suggests that the magnetic interactions between 4f metal ions are even stronger than in **1** which stays in good agreement with structural data indicating shorter Dy–Dy distances in **2**. Thus, to examine the intrinsic single-ion anisotropy of Dy^III^ in **2**, we investigated the *ac* magnetic properties for the Y^III^-diluted sample of **2@Y** (Figs. 2 and S10). Thanks to the magnetic dilution effect, **2@Y** exhibits the field-induced slow magnetic relaxation under very similar field and temperature conditions as observed in **1@Y**. However, the relaxation processes are slightly accelerated which results in the observation of only the onset of slow magnetic relaxation in the high-frequency region (Fig. 2ii, iv). Therefore, it was not possible to apply the generalized Debye model to fit the *ac* magnetic curves and determine the related magnetic relaxation times. We only applied the simplified approach based on the linear parts of the ln(*χ*_M_”/*χ*_M_’) versus *T*^–1^ plots (Fig. 2vi)^103,104^. These dependencies were linear in the whole accessible temperature range related to the onsets of the *χ*_M_” signal enabling the determination of the effective energy barrier of the Arrhenius-type thermal relaxation pathway. The resulting *U*_eff_ of 20.1(3) K is comparable with the energy barrier of 26.4(5) K for Orbach relaxation in **1@Y** suggesting the analogous single-ion origin of slow magnetic relaxation. Its smaller value indicates that the intrinsic magnetic anisotropy of Dy^III^ in **2** is weaker than in **1**. This can be correlated with more axial character of the 4f metal complexes in **1** which can be related to its lower crystal symmetry (Table 2). Even that the magnetic dilution in **1@Y** and **2@Y** improves the slow magnetic relaxation effects, the magnetic hysteresis at the lowest accessible temperature of 1.8 K is not observed for any of the magnetically diluted samples (Figure S2) which is related to very weak single-ion anisotropy of investigated Dy^III^ centers. We also checked **3** and **4** for the SMM behavior but *ac* measurements point out they do not exhibit SMM features that can be assigned to short distances between Dy^III^ centers and higher unit cell symmetry.

For the additional proof for the appearance of magnetic anisotropy in organic Dy^III^ borohydrides of **1** and **2**, we performed the ab initio calculations of CASSCF/RASSI/SINGLE_ANISO type which are currently the most powerful computational tool for the analysis of single-ion anisotropy of lanthanide molecular nanomagnets (see the computational details in the Supporting Information)^28,29,106^. The ab initio calculations were performed on the experimental crystal structures of **1**–**4** consisting of the Dy^III^ centers with the directly attached borohydride ions, four in the case of **1**–**2**, and six in the case of **3**–**4** (Figure S11). For comparative analysis within the whole series of **1**–**4**, two different basis sets, small **S** and large **L**, differing in the basis function qualities, were employed (Table S3). The obtained results are gathered in Tables S4–S7. From these results, it is clearly seen that **1** exhibits a distinct single-ion anisotropy of Dy^III^ centers represented by the axial ground Kramers doublet with the high *g*_z_ value of ca. 19.5 is which characteristic for the $|+15/2\rangle$ *m*_J_ state dominant within the ground multiplet (Table S4). The magnetic easy axis of the ground multiplet is situated nearly along the Dy–B direction related to the borohydride revealing the closest distance to the metal ion (Figure S11) which indicates that the asymmetric arrangement of borohydride ions and their variable distances to the 4f metal ion are responsible for the induction of lanthanide magnetic anisotropy. It is, however, imperfect as there is a non-negligible admixture of other *m*_J_ states to the ground doublet which gives rise to the *g*_x_, *g*_y_ values above 0.025. As a result, the strong QTM effect is generated which finds the reflection in the *ac* magnetic data showing that the SMM character is observed only under the applied *dc* magnetic field (Fig. 2). The first excited Kramers doublet is lying ca. 100 cm^–1^ above and reveals the much less axial character, thus it can be involved in the Orbach relaxation pathway. However, its energy position is much higher than the experimental energy barrier of 26.4(5) K (18.4(4) cm^–1^) found for the contribution from an Orbach process. This can be only partially ascribed to the underestimation of the experimental energy barrier due to the unresolved admixture of Raman relaxation process (see above). Thus, this difference can be mainly explained by the uncertainty of the critical Dy–B and Dy–H distances in the crystal structure used for the ab initio calculations as it comes from the PXRD analysis, which is much less accurate that the structural models from the single-crystal X-ray analyses, usually employed for this type of calculations^106^. Nevertheless, the generation of a significant single-ion anisotropy of Dy^III^ centers in **1** is supported by the obtained results of the ab initio calculations. This is particularly depicted by the comparison with the computed Dy^III^ crystal-field effects in the highly symmetrical inorganic Dy^III^ borohydride complexes of **3** and **4** (Tables S6–S7). In the large basis **L**, both **3** and **4** exhibit an easy-plane type of Dy^III^ magnetic anisotropy on the ground Kramers doublet characterized by the highly mixed *m*_J_ composition. This agrees well with the lack of *ac* magnetic signal in these two compounds and can be straightforwardly correlated with the high symmetry of the related Dy^III^ complexes with six closely positioned borohydride anions (Figure S11). We also performed the ab initio calculations for Dy^III^ complexes in **2** (Table S5). The resulting ground Kramers doublet is highly mixed leading to the non-axial character (Fig. 2). These results are not changed even for the more precise calculations using the additionally enlarged basis sets (Tables S8–S9). This indicates a much worse single-ion anisotropy for **2** than observed in **1** which can be correlated with the presence of four identical Dy–B distances in **2** while four different Dy–B distances detected in **1** (Fig. 1), and stays in a good agreement with the experiment showing only the onset of slow magnetic relaxation on **2** even under the optimal *dc* field (Fig. 2). It suggests that the deformation of Dy^III^ complexes towards an asymmetric arrangement of borohydrides in **1** plays a critical role, more important than the generally decreased crystal symmetry occurring going from **3**–**4** to **1** and **2**.

### Luminescence properties

**Figure 3:**
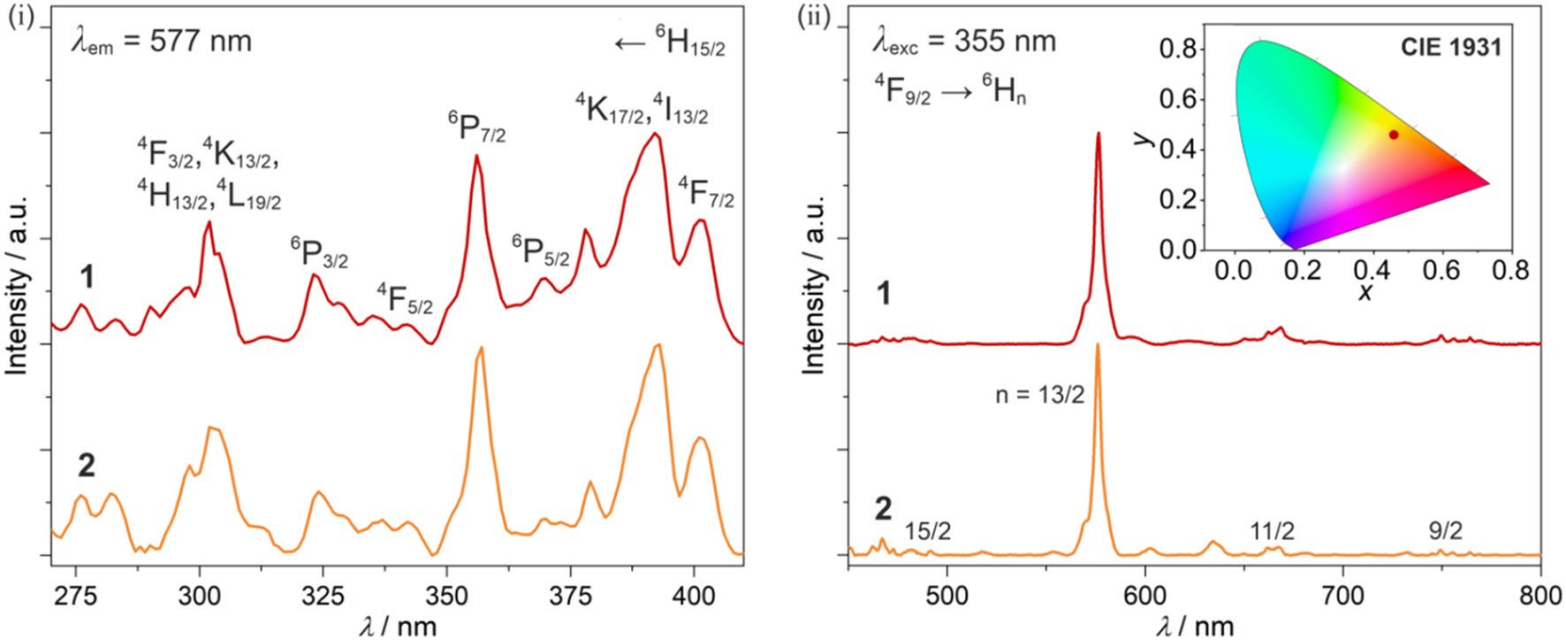
Solid-state room-temperature photoluminescence of **1** and **2**: excitation spectra for the emission at 577 nm with the indicated f-f electronic transitions (i), and the respective emission spectra for the excitation wavelength of 355 nm with the indicated f–f electronic transitions (ii), together with the resulting color (identical for **1** and **2**) presented on the CIE 1931 chromaticity diagram shown in the inset.

Due to the very weak visible light absorption, and the intrinsic photoluminescent character of Dy^3+^ ions, we investigated solid-state UV-induced emission properties of **1** and **2** at both room and liquid nitrogen temperatures (Figs. 3 and S12). At 298 K, under the UV light irradiation, **1** and **2** exhibit yellow emission mainly related to the strong sharp peak centered at 576 nm (Fig. 3ii). This dominant emission band is accompanied by a series of much weaker peaks at ca. 490, 665, and 750 nm. All of them can be assigned to the f-f electronic transitions of Dy^III^ occurring from the emissive ^4^F_9/2_ level to the lower-lying ^6^H_15/2, 13/2, 11/2, 9/2_ multiplets^25^. It results in the overall yellow emission depicted by the *xy* CIE 1931 chromaticity parameters of (0.46, 0.46), almost identical for **1** and **2** (Fig. 3). The rich excitation patterns (Fig. 3i), consisting of the series of sharp peaks rather than the broad bands, indicate that the emission properties are due to the direct f–f excitation pathways as these excitation peaks can be assigned to the specific electronic transitions from the ground ^6^H_15/2_ multiplet to the higher-lying excited states within the Dy^III^ centers^107^. There is no proof for an intermolecular energy transfer process which could be expected particularly for **2** incorporating expanded Ph_4_P^+^ cations. Nevertheless, yellow photoluminescence both in **1** and **2** was detected at room temperature. It remains almost unchanged on cooling to the liquid nitrogen (Figure S12), again suggesting that the additional energy transfer processes are not observed and the luminescence can be ascribed to the single-ion property of Dy^3+^ ions. As a result, the Y^III^-diluted samples were not expected to produce extra emission features, except of the emission intensity variation which is difficult to discuss due to the overall weak emission signal. Thus, the **1@Y** and **2@Y** were not investigated. On the other hand, we checked luminescent properties in **3** and **4**. Emission of **3** has an extremely low intensity, on the noise level. As for **4**, with even higher symmetry, no emission signal was observed. This can be assigned to shorter distances between Dy^III^ centers than in **1** and **2** which leads to the mutual emission quenching.

## Conclusions

We report the successful mechanochemical synthesis of [Cat][RE(BH_4_)_4_], [Cat] = n-Bu_4_N, Ph_4_P, for RE = Dy, Y_0.9_Dy_0.1_, together with the crystal structures, luminescence, and magnetic properties of the obtained materials. The compound with n-Bu_4_N^+^ ion crystallizes in a monoclinic unit cell, in the space group *P*2_1_/c, while the system with Ph_4_P^+^ ion has a significantly higher symmetry, adopting a tetragonal unit cell (*I*4_1_/a). Both magnetic and luminescent properties of [n-Bu_4_N][Dy(BH_4_)_4_] and [Ph_4_P][Dy(BH_4_)_4_] were compared with those for α/β-Dy(BH_4_)_3_ phases crystallizing in cubic unit cells. The organic borohydride derivatives reveal the Single-Molecule-Magnet behavior, presented for the first time for the derivatives of RE(BH_4_)_3_, which was illustrated for their magnetically Y^III^-diluted analogs, [n-Bu_4_N][Y_0.9_Dy_0.1_(BH_4_)_4_] and [Ph_4_P][Y_0.9_Dy_0.1_(BH_4_)_4_]. The most pronounced single-ion anisotropy of Dy^III^ complexes was observed in the lowest symmetrical phase of [n-Bu_4_N][Dy(BH_4_)_4_] which was confirmed by the results of the ab initio calculations performed along the whole series of discussed compounds. Moreover, the obtained organic Dy(III) borohydrides serve as luminescent molecular nanomagnets, which combine room-temperature lanthanide-based photoluminescence in the solid-state with a field-induced slow magnetic relaxation effect. Their yellow luminescence was achievable due to the absence of solvent molecules and colored chromophore components, as well as the sufficient separation of 4f metal ions within the crystal lattice. This interpretation is supported by the investigation of α/β-Dy(BH_4_)_3_ phases which do not reveal any luminescent features. They are also magnetically isotropic without any sign of the SMM character. It means that Dy^III^ magnetic anisotropy is generated by the low-symmetry coordination environment observed in the organic derivatives. Therefore, this work shows a simple route to luminescent SMMs realized by the insertion of well-known organic cations, tetrabutylammonium and tetraphenylphosphonium, into Dy(III) borohydrides. Such simple modification of classical inorganic borohydrides was found to be efficient in inducing attractive optical and magnetic properties. Future work can be focused on the further functionalization of lanthanide(III) borohydrides using chiral and/or polar organic cations which can enrich multifunctional potential towards chiral or ferroelectric luminescent SMMs.

## Supplementary Information

Supplementary Information.

## Data Availability

The datasets generated during and/or analyzed during the current study are available from the corresponding author on reasonable request.

## References

[1] Craig GA, Murrie M (2015). 3d single-ion magnets. Chem. Soc. Rev..

[2] Ishikawa N, Sugita M, Ishikawa T, Koshihara SY, Kaizu Y (2003). Lanthanide double-decker complexes functioning as magnets at the single-molecular level. J. Am. Chem. Soc..

[3] Novak MA, Sessoli R, Gatteschi D, Caneschi A (1993). Magnetic bistability in a metal-ion cluster. Nature.

[4] Demir S, Gonzalez MI, Darago LE, Evans WJ, Long JR (2017). Giant coercivity and high magnetic blocking temperatures for N_2_^3−^ radical-bridged dilanthanide complexes upon ligand dissociation. Nat. Commun..

[5] Mannini M (2009). Magnetic memory of a single-molecule quantum magnet wired to a gold surface. Nat. Mater..

[6] Studniarek M (2019). Understanding the superior stability of single-molecule magnets on an oxide film. Adv. Sci..

[7] Bogani L, Wernsdorfer W (2008). Molecular spintronics using single-molecule magnets. Nat. Mater..

[8] Moreno-Pineda E, Godfrin C, Balestro F, Wernsdorfer W, Ruben M (2018). Molecular spin qudits for quantum algorithms. Chem. Soc. Rev..

[9] Gaita-Ariño A, Luis F, Hill S, Coronado E (2019). Molecular spins for quantum computation. Nat. Chem..

[10] Woodruff DN, Winpenny REP, Layfield RA (2013). Lanthanide single-molecule magnets. Chem. Rev..

[11] Blagg RJ (2013). Magnetic relaxation pathways in lanthanide single-molecule magnets. Nat. Chem..

[12] Liu J (2016). A stable pentagonal bipyramidal Dy(III) single-ion magnet with a record magnetization reversal barrier over 1000 K. J. Am. Chem. Soc..

[13] Goodwin CAP, Ortu F, Reta D, Chilton NF, Mills DP (2017). Molecular magnetic hysteresis at 60 kelvin in dysprosocenium. Nature.

[14] Liu J-L, Chen Y-C, Tong M-L (2018). Symmetry strategies for high performance lanthanide-based single-molecule magnets. Chem. Soc. Rev..

[15] Chen Y-C (2017). Hyperfine-interaction-driven suppression of quantum tunneling at zero field in a holmium(III) single-ion magnet. Angew. Chem. Int. Ed..

[16] Guo F-S (2018). Magnetic hysteresis up to 80 kelvin in a dysprosium metallocene single-molecule magnet. Science.

[17] Bünzli JCG, Piguet C (2005). Taking advantage of luminescent lanthanide ions. Chem. Soc. Rev..

[18] Jia J-H, Li Q-W, Chen Y-C, Liu J-L, Tong M-L (2019). Luminescent single-molecule magnets based on lanthanides: Design strategies, recent advances and magneto-luminescent studies. Coord. Chem. Rev..

[19] Li X (2017). A stimuli-responsive smart lanthanide nanocomposite for multidimensional optical recording and encryption. Angew. Chem. Int. Ed..

[20] Moynihan S, Van Deun R, Binnemans K, Redmond G (2007). Optical properties of planar polymer waveguides doped with organo-lanthanide complexes. Opt. Mater..

[21] Bui AT (2017). Near infrared two photon imaging using a bright cationic Yb(III) bioprobe spontaneously internalized into live cells. Chem. Commun..

[22] Yan B (2017). Lanthanide-functionalized metal-organic framework hybrid systems to create multiple luminescent centers for chemical sensing. Acc. Chem. Res..

[23] Hasegawa Y, Kitagawa Y (2019). Thermo-sensitive luminescence of lanthanide complexes, clusters, coordination polymers and metal–organic frameworks with organic photosensitizers. J. Mater. Chem. C.

[24] Pointillart F, le Guennic B, Cador O, Maury O, Ouahab L (2015). Lanthanide ion and tetrathiafulvalene-based ligand as a “magic” couple toward luminescence, single molecule magnets, and magnetostructural correlations. Acc. Chem. Res..

[25] Chorazy S, Rams M, Nakabayashi K, Sieklucka B, Ohkoshi S (2016). White light emissive Dy^III^ single-molecule magnets sensitized by diamagnetic [Co^III^(CN)_6_]^3−^ linkers. Chem. A Eur. J..

[26] Bi Y (2016). Thermostability and photoluminescence of Dy(III) single-molecule magnets under a magnetic field. Chem. Sci..

[27] Errulat D (2019). A luminescent thermometer exhibiting slow relaxation of the magnetization: toward self-monitored building blocks for next-generation optomagnetic devices. ACS Cent. Sci..

[28] Wang J (2020). Proton conductive luminescent thermometer based on near-infrared emissive YbCo_2_ molecular nanomagnets. J. Am. Chem. Soc..

[29] Wang J (2021). Holmium(III) molecular nanomagnets for optical thermometry exploring the luminescence re-absorption effect. Chem. Sci..

[30] Long J (2015). A high-temperature molecular ferroelectric Zn/Dy complex exhibiting single-ion-magnet behavior and lanthanide luminescence. Angew. Chem. Int. Ed..

[31] Chorazy S, Kumar K, Nakabayashi K, Sieklucka B, Ohkoshi S (2017). Fine tuning of multicolored photoluminescence in crystalline magnetic materials constructed of trimetallic Eu_x_Tb_1–x_[Co(CN)_6_] cyanido-bridged chains. Inorg. Chem..

[32] Chorazy S, Zychowicz M, Ohkoshi S, Sieklucka B (2019). Wide-range UV-to-visible excitation of near-infrared emission and slow magnetic relaxation in Ln^III^ (4,4′-Azopyridine-1,1′-dioxide)[Co^III^(CN)_6_]^3–^ layered frameworks. Inorg. Chem..

[33] Brunet G (2019). Exploring the dual functionality of an ytterbium complex for luminescence thermometry and slow magnetic relaxation. Chem. Sci..

[34] Ma S, Zhang T, Zhao J-P, Liu Z-Y, Liu F-C (2021). A magnetic site dilution approach to achieve bifunctional fluorescent thermometers and single-ion magnets. Dalton Trans..

[35] Speed S (2017). Lanthanide complexes involving multichelating TTF-based ligands. Inorg. Chem. Front..

[36] Liu C-M, Zhang D-Q, Hao X, Zhu D-B (2017). A Chinese pane-like 2D metal-organic framework showing magnetic relaxation and luminescence dual-functions. Sci. Rep..

[37] Kalinke LHG (2019). Metal-organic frameworks as playgrounds for reticulate single-molecule magnets. Inorg. Chem..

[38] Xin Y (2019). Dehydration-hydration switching of single-molecule magnet behavior and visible photoluminescence in a cyanido-bridged Dy^III^Co^III^ framework. J. Am. Chem. Soc..

[39] Zakrzewski JJ, Chorazy S, Nakabayashi K, Ohkoshi S, Sieklucka B (2019). Photoluminescent lanthanide(III) Single-Molecule Magnets In Three-Dimensional Polycyanidocuprate(I)-Based Frameworks. Chem. A Eur. J..

[40] Fan K (2020). Luminescent Ir(III)–Ln(III) coordination polymers showing slow magnetization relaxation. Inorg. Chem. Front..

[41] Schlesinger HI, Sanderson RT, Burg AB (1939). A volatile compound of aluminum, boron and hydrogen. J. Am. Chem. Soc..

[42] Schlesinger HI, Brown HC (1953). Uranium(IV) borohydride. J. Am. Chem. Soc..

[43] Ephritikhine M (1997). Synthesis, structure, and reactions of hydride, borohydride, and aluminohydride compounds of the f-elements. Chem. Rev..

[44] Schlesinger HI, Brown HC, Hoekstra HR, Rapp LR (1953). Reactions of diborane with alkali metal hydrides and their addition compounds. New syntheses of borohydrides. Sodium and potassium borohydrides. J. Am. Chem. Soc..

[45] Schlesinger HI, Brown HC, Hyde EK (1953). The preparation of other borohydrides by metathetical reactions utilizing the alkali metal borohydrides. J. Am. Chem. Soc..

[46] Nora de Souza MV, Alves Vasconcelos TR (2006). Recent methodologies mediated by sodium borohydride in the reduction of different classes of compounds. Appl. Organomet. Chem..

[47] Grochala W, Edwards PP (2004). Thermal decomposition of the non-interstitial hydrides for the storage and production of hydrogen. Chem. Rev..

[48] Bannenberg LJ (2020). Metal (boro-) hydrides for high energy density storage and relevant emerging technologies. Int. J. Hydrog. Energy.

[49] Rivard E, Trudeau M, Zaghib K (2019). Hydrogen storage for mobility: a review. Materials (Basel).

[50] Rude LH (2011). Tailoring properties of borohydrides for hydrogen storage: a review. Phys. Status Solidi.

[51] Nakamori, Y. & Orimo, S. Borohydrides as hydrogen storage materials. in *Solid-State Hydrogen Storage* 420–449 (Elsevier, 2008).

[52] Li H-W, Yan Y, Orimo S, Züttel A, Jensen CM (2011). Recent progress in metal borohydrides for hydrogen storage. Energies.

[53] Orimo S, Nakamori Y, Eliseo JR, Züttel A, Jensen CM (2007). Complex hydrides for hydrogen storage. Chem. Rev..

[54] Churchard AJ (2011). A multifaceted approach to hydrogen storage. Phys. Chem. Chem. Phys..

[55] Cuan J (2019). Borohydride-Scaffolded Li/Na/Mg fast ionic conductors for promising solid-state electrolytes. Adv. Mater..

[56] Gulino V (2019). Phase Stability And Fast Ion Conductivity In The Hexagonal LiBH_4_-LiBr-LiCl solid solution. Chem. Mater..

[57] GharibDoust SP (2017). Synthesis, structure, and li-ion conductivity of LiLa(BH_4_)_3_X, X = Cl, Br, I. J. Phys. Chem. C.

[58] Zhang T (2018). Ammonia, a switch for controlling high ionic conductivity in lithium borohydride ammoniates. Joule.

[59] Fadlallah S (2020). Rationalizing the reactivity of mixed allyl rare-earth borohydride complexes with DFT studies. Catalysts.

[60] Visseaux M, Bonnet F (2011). Borohydride complexes of rare earths, and their applications in various organic transformations. Coord. Chem. Rev..

[61] Wegner W, Jaroń T, Grochala W (2018). Preparation of a series of lanthanide borohydrides and their thermal decomposition to refractory lanthanide borides. J. Alloys Compd..

[62] Jensen JA, Gozum JE, Pollina DM, Girolami GS (1988). Titanium, zirconium, and hafnium tetrahydroborates as ‘tailored’ CVD precursors for metal diboride thin films. J. Am. Chem. Soc..

[63] Zavorotynska O, El-Kharbachi A, Deledda S, Hauback BC (2016). Recent progress in magnesium borohydride Mg(BH_4_)_2_: Fundamentals and applications for energy storage. Int. J. Hydrog. Energy.

[64] Wegner W (2016). Organic derivatives of Mg(BH_4_)_2_ as precursors towards MgB_2_ and novel inorganic mixed-cation borohydrides. Dalton Trans..

[65] Yan Y (2015). A novel strategy for reversible hydrogen storage in Ca(BH_4_)_2_. Chem. Commun..

[66] Wegner W, Fijalkowski KJ, Grochala W (2020). A low temperature pyrolytic route to amorphous quasi-hexagonal boron nitride from hydrogen rich (NH_4_)_3_Mg(BH_4_)_5_. Dalton Trans..

[67] Frommen C (2010). Crystal structure, polymorphism, and thermal properties of yttrium borohydride Y(BH_4_)_3_. J. Alloys Compd..

[68] Sato T (2008). Experimental and computational studies on solvent-free rare-earth metal borohydrides R(BH_4_)_3_ (R = Y, Dy, and Gd). Phys. Rev. B.

[69] Ley MB, Jørgensen M, Černý R, Filinchuk Y, Jensen TR (2016). From M(BH_4_)_3_ (M = La, Ce) borohydride frameworks to controllable synthesis of porous hydrides and ion conductors. Inorg. Chem..

[70] GharibDoust SP (2018). Synthesis, structure, and polymorphic transitions of praseodymium(III) and neodymium(III) borohydride, Pr(BH_4_)_3_ and Nd(BH_4_)_3_. Dalton Trans..

[71] Grinderslev JB, Møller KT, Bremholm M, Jensen TR (2019). Trends in synthesis, crystal structure, and thermal and magnetic properties of rare-earth metal borohydrides. Inorg. Chem..

[72] Olsen JE (2014). Structure and thermal properties of composites with RE-borohydrides (RE = La, Ce, Pr, Nd, Sm, Eu, Gd, Tb, Er, Yb or Lu) and LiBH_4_. RSC Adv..

[73] Wegner W, Jaroń T, Grochala W (2014). Polymorphism and hydrogen discharge from holmium borohydride, Ho(BH_4_)_3_, and KHo(BH_4_)_4_. Int. J. Hydrog. Energy.

[74] Gennari FC (2013). Mechanochemical synthesis of erbium borohydride: Polymorphism, thermal decomposition and hydrogen storage. J. Alloys Compd..

[75] Olsen JE, Frommen C, Sørby MH, Hauback BC (2013). Crystal structures and properties of solvent-free LiYb(BH_4_)_4−x_Cl_x_, Yb(BH_4_)_3_ and Yb(BH_4_)_2−x_Cl_x_. RSC Adv..

[76] Humphries TD (2015). Crystal structure and in situ decomposition of Eu(BH_4_)_2_ and Sm(BH_4_)_2_. J. Mater. Chem. A.

[77] Richter B, Grinderslev JB, Møller KT, Paskevicius M, Jensen TR (2018). From metal hydrides to metal borohydrides. Inorg. Chem..

[78] Christmann J, Mansouri A, Grinderslev JB, Jensen TR, Hagemann H (2020). Probing the local symmetry of Tb^3+^ in borohydrides using luminescence spectroscopy. J. Lumin..

[79] Schouwink P (2014). Structure and properties of complex hydride perovskite materials. Nat. Commun..

[80] Wegner W, van Leusen J, Majewski J, Grochala W, Kögerler P (2019). Borohydride as magnetic superexchange pathway in late lanthanide borohydrides. Eur. J. Inorg. Chem..

[81] Jaroń T, Wegner W, Cyrański MK, Grochala W (2012). Tetrabutylammonium cation in a homoleptic environment of borohydride ligands: [(n-Bu)_4_N][BH_4_] and [(n-Bu)_4_N][Y(BH_4_)_4_]. J. Solid State Chem..

[82] Starobrat A, Jaroń T, Grochala W (2015). Synthesis and characterization of a series of mixed-cation borohydrides of scandium: [Cat][Sc(BH_4_)_4_], [Cat] = [Me_4_N], [n-Bu_4_N], and [Ph_4_P]. Inorg. Chim. Acta.

[83] Antsyshkina AS, Sadikov GG, Borisov P, Makhaev VD (2001). Complexes of yttrium, thulium, and lutetium tetrahydridoborates with tetraphenylphosphonium tetrahydridoborate (Ph_4_P)[M(BH_4_)_4_] (M = Y, Tm, Lu): Crystal structure of (Ph_4_P)[Tm(BH_4_)_4_]. Russ. J. Inorg. Chem..

[84] Eliseeva SV, Salerno EV, Lopez Bermudez BA, Petoud S, Pecoraro VL (2020). Dy^3+^ white light emission can be finely controlled by tuning the first coordination sphere of Ga^3+^/Dy^3+^ metallacrown complexes. J. Am. Chem. Soc..

[85] Petricek V, Dusek M, Palatinus L (2014). Crystallographic computing system JANA2006: General features. Z. Krist..

[86] Momma K, Izumi F (2011). VESTA 3 for three-dimensional visualization of crystal, volumetric and morphology data. J. Appl. Crystallogr..

[87] Černý R, Schouwink P (2015). The crystal chemistry of inorganic metal boro-hydrides and their relation to metal oxides. Acta Crystallogr. Sect. B Struct. Sci. Cryst. Eng. Mater..

[88] Wegner W, Jaroń T (2021). Synthesis, polymorphism and thermal decomposition process of (n-C_4_H_9_)_4_N*RE*(BH_4_)_4_ for *RE* = Ho, Tm and Yb. Materials.

[89] Hagemann H (2008). LiSc(BH4)4: A novel salt of Li+ and Discrete Sc(BH4)4−complex anions. J. Phys. Chem. A.

[90] Černý R (2010). NaSc(BH_4_)_4_: A novel scandium-based borohydride. J. Phys. Chem. C.

[91] Černý R (2010). Structure and characterization of KSc(BH_4_)_4_. J. Phys. Chem. C.

[92] Starobrat A, Jaroń T, Grochala W (2018). New hydrogen-rich ammonium metal borohydrides, NH_4_[M(BH_4_)_4_], M = Y, Sc, Al, as potential H _2_ sources. Dalton Trans..

[93] Starobrat A, Jaroń T, Grochala W (2019). Two new derivatives of scandium borohydride, MSc(BH_4_)_4_, M = Rb, Cs, prepared via a one-pot solvent-mediated method. Dalton Trans..

[94] Jaroń T, Wegner W, Fijałkowski KJ, Leszczyński PJ, Grochala W (2015). Facile formation of thermodynamically unstable novel borohydride materials by a wet chemistry route. Chem. A Eur. J..

[95] Jaroń T, Grochala W (2011). Probing Lewis acidity of Y(BH_4_)_3_ via its reactions with MBH_4_ (M = Li, Na, K, NMe_4_). Dalton Trans..

[96] Jaroń T, Wegner W, Grochala W (2013). M[Y(BH_4_)_4_] and M_2_Li[Y(BH_4_)_6−x_Cl_x_] (M = Rb, Cs): New borohydride derivatives of yttrium and their hydrogen storage properties. Dalton Trans..

[97] Gharibdoust SHP, Ravnsbæk DB, Černý R, Jensen TR (2017). Synthesis, structure and properties of bimetallic sodium rare-earth (RE) borohydrides, NaRE(BH_4_)_4_, RE = Ce, Pr, Er or Gd. Dalton Trans..

[98] Heere M (2017). In situ investigations of bimetallic potassium erbium borohydride. Int. J. Hydrog. Energy.

[99] Wegner W, Jaroń T, Grochala W (2013). MYb(BH_4_)_4_ (M = K, Na) from laboratory X-ray powder data. Acta Crystallogr. Sect. C Cryst. Struct. Commun..

[100] Chorazy S, Wang J, Ohkoshi SI (2016). Yellow to greenish-blue colour-tunable photoluminescence and 4f-centered slow magnetic relaxation in a cyanido-bridged Dy^III^(4-hydroxypyridine)-Co^III^ layered material. Chem. Commun..

[101] Wu J (2017). Axial ligand field in D4d coordination symmetry: magnetic relaxation of Dy SMMs perturbed by counteranions. Inorg. Chem..

[102] Arauzo A (2020). Coumarin-lanthanide based compounds with SMM behavior and high quantum yield luminescence. Dalton Trans..

[103] Ferrando-Soria J (2011). Rational enantioselective design of chiral heterobimetallic single-chain magnets: Synthesis, crystal structures and magnetic properties of oxamato-bridged M^II^Cu^II^ chains (M=Mn, Co). Chem. A Eur. J..

[104] Chorazy S (2012). Conjunction of chirality and slow magnetic relaxation in the supramolecular network constructed of crossed cyano-bridged Co^II^-W^V^ molecular chains. J. Am. Chem. Soc..

[105] Liddle ST, Van Slageren J (2015). Improving f-element single molecule magnets. Chem. Soc. Rev..

[106] Ungur L, Chibotaru LF (2016). Ab initio crystal field for lanthanides. Chem. Eur. J..

[107] Kofod N, Arppe-Tabbara R, Sørensen TJ (2019). Electronic energy levels of dysprosium(III) ions in solution. Assigning the emitting state and the intraconfigurational 4f–4f transitions in the vis-NIR Region And Photophysical Characterization of Dy(III) in water, methanol, and dimethyl sulfoxide. J. Phys. Chem. A.

