# Supplementary material for: Incorporation of expanded organic cations in dysprosium(III) borohydrides for achieving luminescent molecular nanomagnets
Source: Sci Rep. 2021 May 31;11:11354. doi: 10.1038/s41598-021-88446-7 (PMC8166919; doi:10.1038/s41598-021-88446-7)
Supplement: Supplementary file 1 — Supplementary Information. [file 41598_2021_88446_MOESM1_ESM.pdf]

SUPPORTING INFORMATION

Incorporation of expanded organic cations in dysprosium(III) borohydrides for achieving luminescent molecular nanomagnets

Wojciech Wegner,[1,2,*] Jakub J. Zakrzewski,[3] Mikolaj Zychowicz,[3] and Szymon Chorazy[3,*]

[1]College of Inter-Faculty Individual Studies in Mathematics and Natural Sciences, University of Warsaw, Banacha 2c, 02-097 Warsaw, Poland

[2]Centre of New Technologies, University of Warsaw, Banacha 2c, 02-097 Warsaw, Poland

[3]Faculty of Chemistry, Jagiellonian University, Gronostajowa 2, 30-387 Krakow, Poland

*chorazy@chemia.uj.edu.pl; w.wegner@cent.uw.edu.pl

Content:

1. Direct-current (*dc*) magnetic properties of **1**, **2**, **1@Y**, and **2@Y**. (Figures S1–S3) 2

2. Alternate-current (*ac*) magnetic properties of **1**, including *dc*-field-variable characteristics at 1.8 K, and temperature-variable characteristics at 1 kOe. (Figure S4) 5

3. Temperature-variable *ac* magnetic characteristics for **1** at H_{dc} = 2.5 kOe. (Figure S5)....6

4. Full *ac* magnetic characteristics of **1@Y** under variable *dc* fields at *T* = 1.8 K. (Figure S6)....7

5. Full temperature-dependent (*ac*) magnetic characteristics of **1@Y** at H_{dc} = 1 kOe. (Figure S7)....8

6. The comparison of the alternative fittings of the temperature dependence of magnetic relaxation time detected for **1@Y** at the optimal *dc* field of 1 kOe. (Figure S8)....9

7. Alternate-current (*ac*) magnetic properties of **2**, including *dc*-field-variable characteristics at 1.8 K, and temperature-variable characteristics at 1 kOe. (Figure S9) 10

8. Full alternate-current (*ac*) magnetic properties of **2@Y**, including *dc*-field-variable characteristics at 1.8 K, and temperature-variable characteristics at 1 kOe. (Figure S10)....11

9. Best-fit parameters for the fittings of temperature- and/or *dc*-field-variable magnetic relaxation times for **1**, **2**, **1@Y**, and **2@Y**. (Table S1) 12

10. Best-fit parameters for various possible fittings of the field- and temperature-dependent magnetic relaxation times in **1@Y**. (Table S2)....13

11. Details of the *ab initio* calculations for Dy^{III} complexes in the crystal structures of **1**–**4** (Tables S3–S9, Figure S11). 14

12. Solid-state low-temperature (77 K) photoluminescent properties of **1** and **2**. (Figure S12)....21

13. Rietveld refinements for **1@Y**, **2@Y**, **1**, and **2**, (Figures S13–S16) and crystal structures of α-$Dy(BH_4)_3$ and β-$Dy(BH_4)_3$ (Figure S17)....22

1. Direct-current (*dc*) magnetic properties of **1**, **2**, **1@Y**, and **2@Y**. (Figures S1–S3)

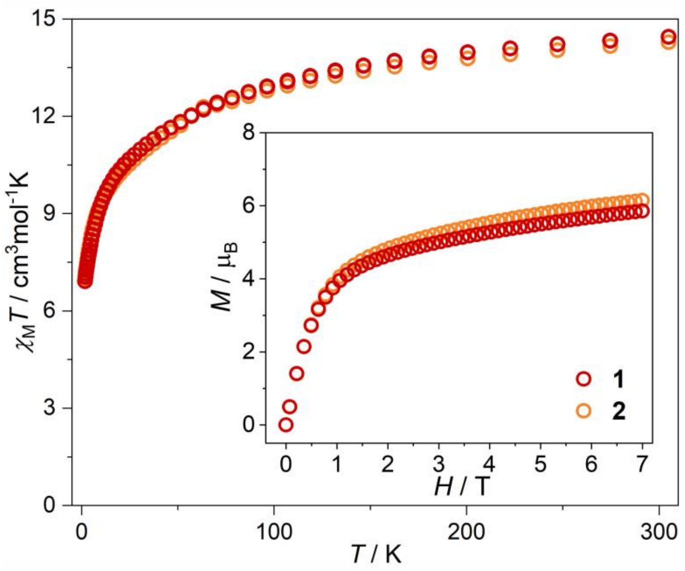

Figure S1. Direct-current (*dc*) magnetic properties of **1** (red points) and **2** (orange points) including the respective temperature dependences of the magnetic susceptibility–temperature product for H_{dc} = 1 kOe, and the related field dependences of magnetization gathered at 1.8 K, shown in the inset.

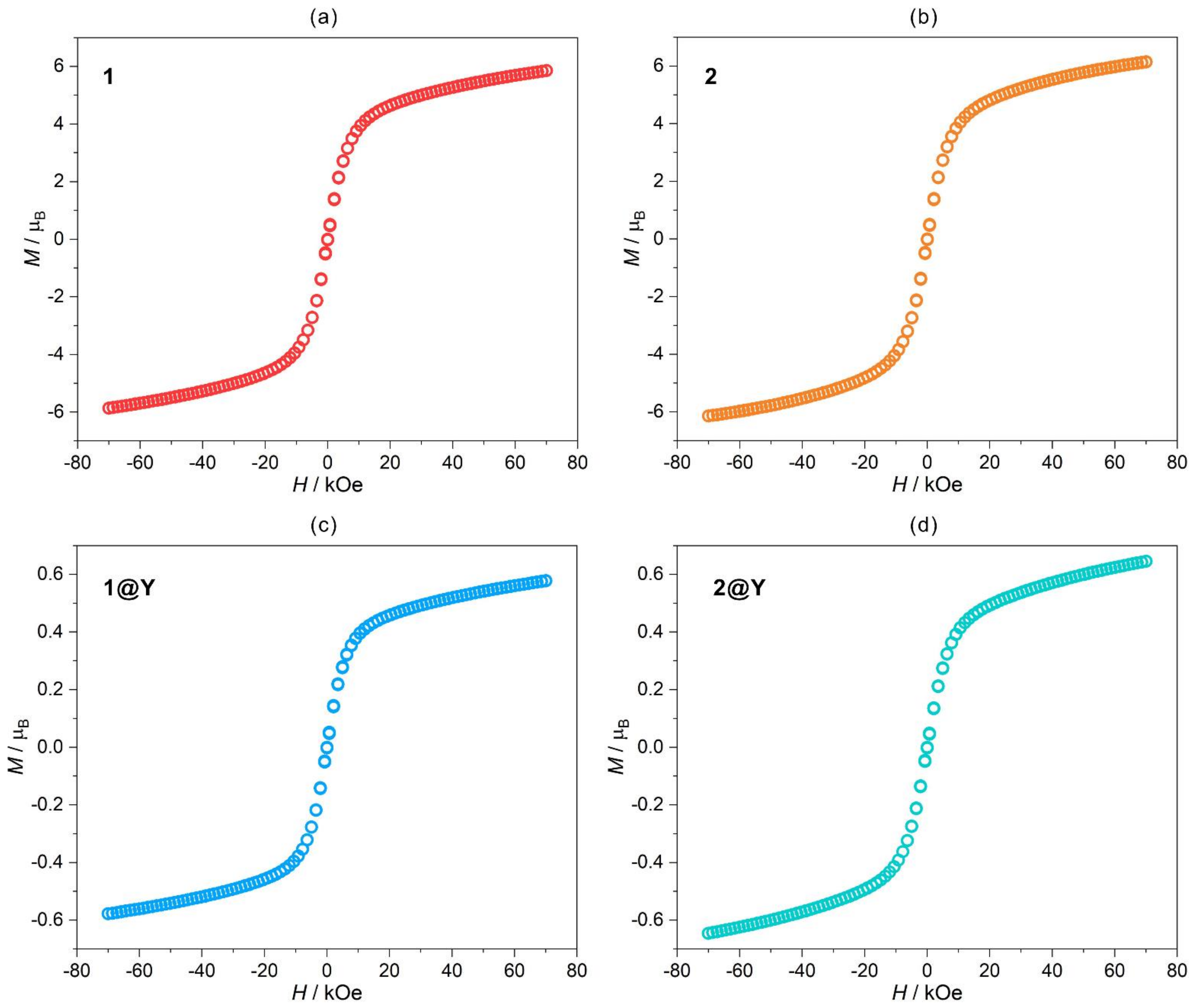

Figure S2. Field dependences of magnetization gathered at T = 1.8 K for the powder samples of **1** (*a*), **2** (*b*), **1@Y** (*c*), and **2@Y** (*d*).

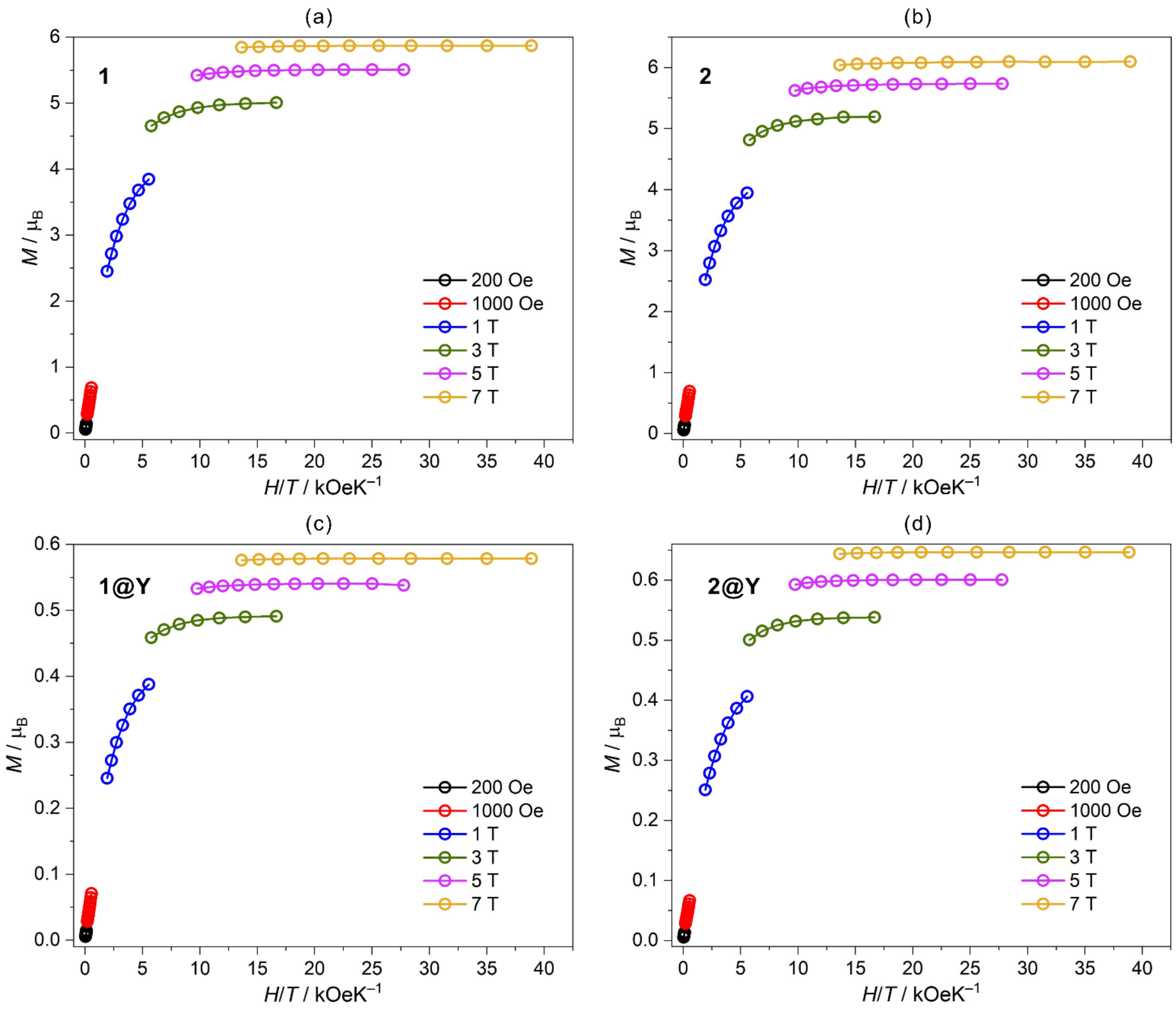

Figure S3. Reduced magnetization curves gathered in the 1.8–5 K range for the powder samples of **1** (*a*), **2** (*b*), **1@Y** (*c*), and **2@Y** (*d*). The solid lines are only to guide the eye.

2. Alternate-current (*ac*) magnetic properties of **1**, including *dc*-field-variable characteristics at 1.8 K, and temperature-variable characteristics at 1 kOe. (Figure S4)

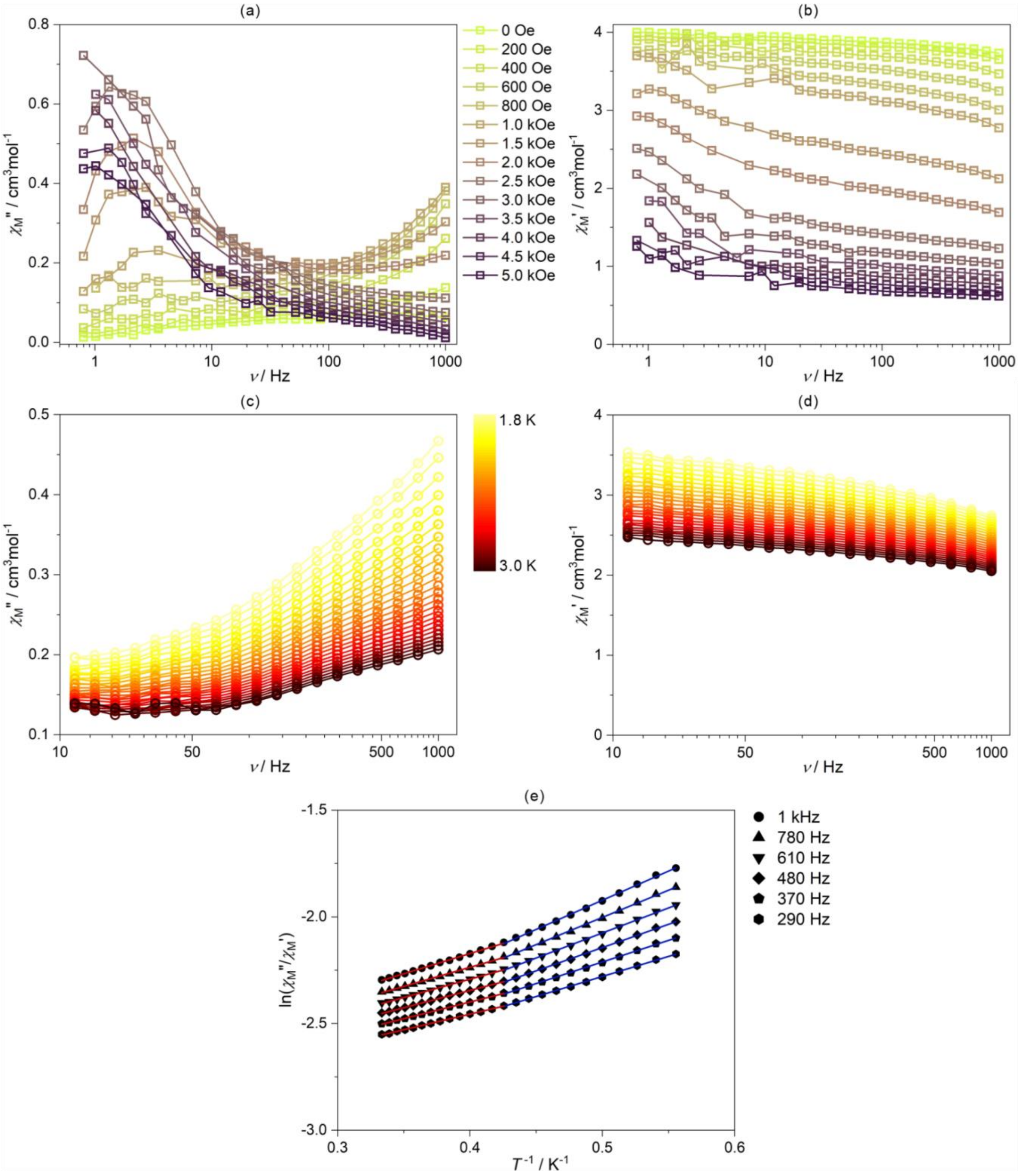

Figure S4. Alternate-current (*ac*) magnetic properties of **1**: *dc*-field-variable frequency dependences of the out-of-phase susceptibility, χ_M" (*a*) and the in-phase susceptibility, χ_M' (*b*) collected in the 0–5 kOe range at T = 1.8 K, temperature-variable frequency dependences of the χ_M" (*c*) and χ_M' (*d*) gathered in the 1.8–3.0 K range at H_{dc} = 1 kOe, and the temperature dependence of the related ln(χ_M"/χ_M') for the indicated frequencies of *ac* field (*e*). The solid lines in (*a*–*d*) are only to guide the eye. In (*e*), the black points represent the experimental data while the solid lines are the best curves for a simplified analysis approach elucidating two different Arrhenius-type relaxation routes operating at lower (blue lines) and higher (red lines) temperatures. The best-fit parameters are gathered in Table S1.

3. Temperature-variable *ac* magnetic characteristics for **1** at H_{dc} = 2.5 kOe. (Figure S5)

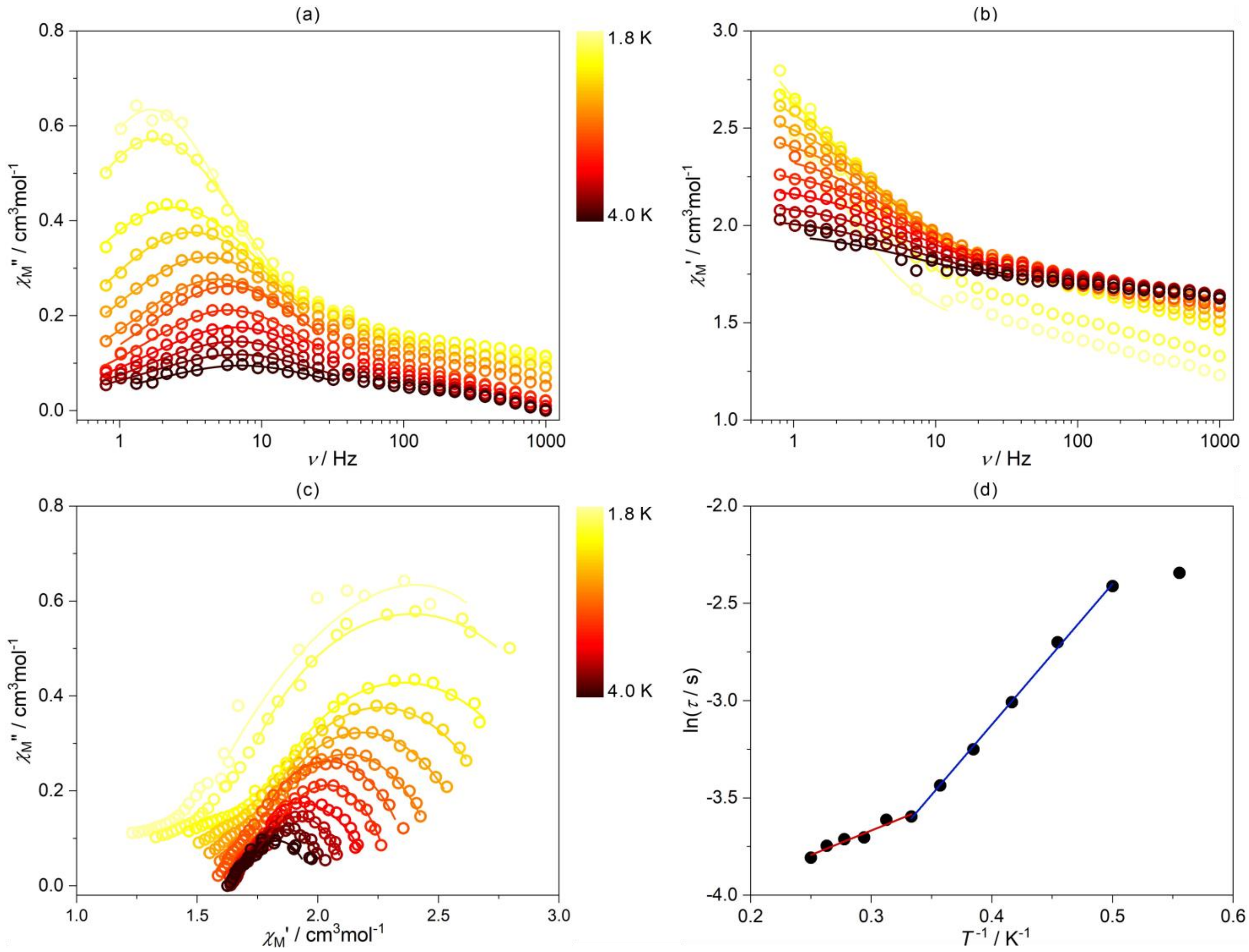

Figure S5. Temperature-variable *ac* magnetic characteristics for **1** at H_{dc} = 2.5 kOe: frequency dependences of the out-of-phase susceptibility, χ_M” (*a*) and the in-phase susceptibility, χ_M’ (*b*) at the indicated temperatures, together with the related Argand plots (*c*), and the resulting temperature dependence of the relaxation time (*d*). The empty points in (*a*–*c*) represent the experimental data while the respective solid lines show the fitting according to the generalized Debye model. The black points in (*d*) represent the experimental data while the solid lines are the best fit-curves for two Arrhenius-type relaxation processes operating in the higher (red line) and lower (blue line) temperature regimes. The best-fit parameters are gathered in Table S1.

4. Full *ac* magnetic characteristics of **1@Y** under variable *dc* fields at T = 1.8 K. (Figure S6)

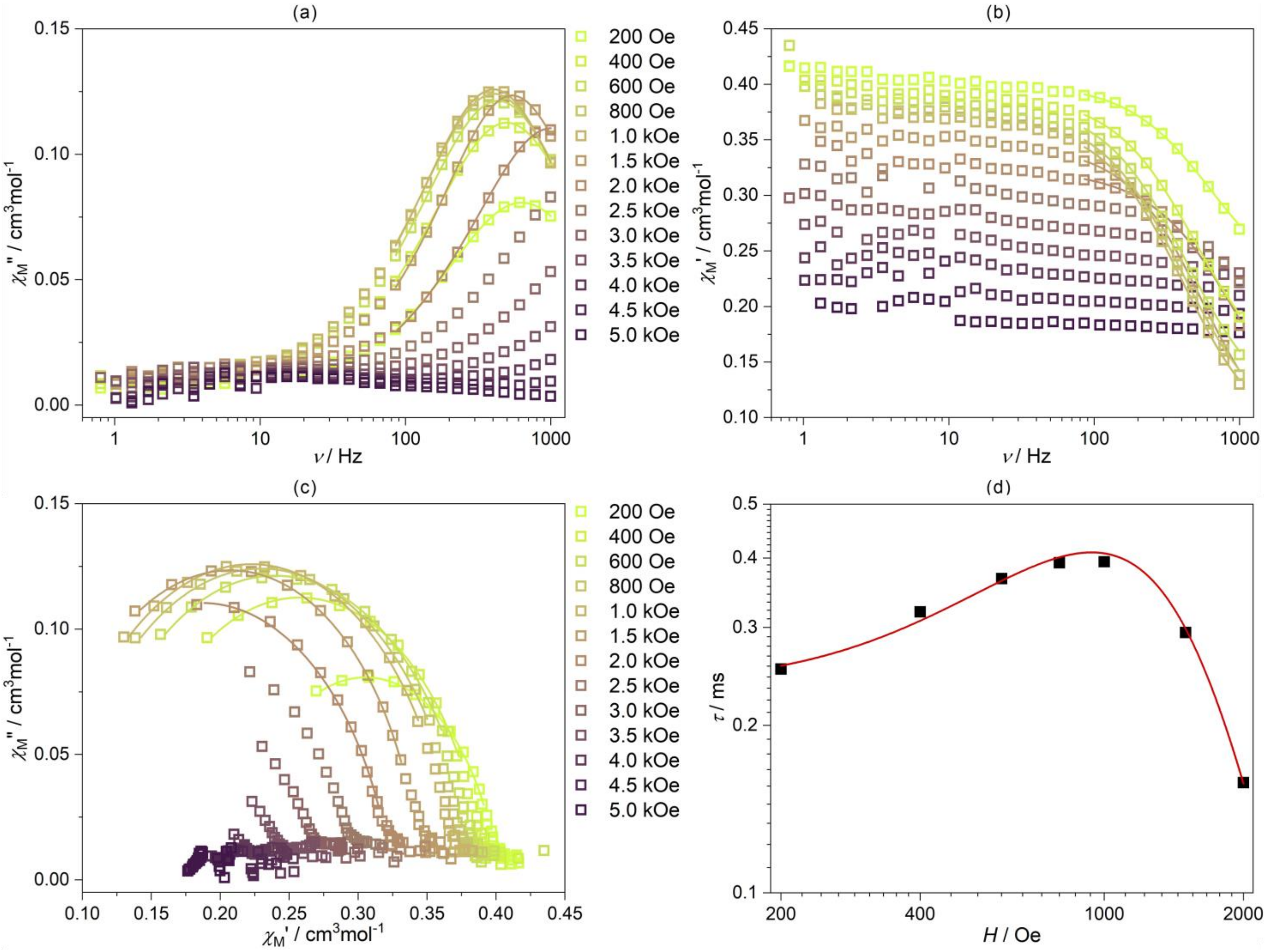

Figure S6. Full alternate-current (*ac*) magnetic characteristics of **1@Y** under variable *dc* magnetic fields at T = 1.8 K (the representative curves are shown in Figure 2): frequency dependences of the out-of-phase magnetic susceptibility, χ_M'' (*a*) and the in-phase susceptibility, χ_M' (*b*) at the indicated *dc* magnetic fields, together with the related Argand plots (*c*), and the resulting field dependence of the relaxation time (*d*). The empty points in (*a*–*c*) represent the experimental data while the respective solid lines show the fitting according to the generalized Debye model. The black points in (*d*) represent the experimental data while the red solid line shows the best-fit curve related to the combined contributions from QTM, direct, and Orbach relaxation processes (the simultaneous fit together with the related temperature-dependence of the relaxation time, Figure S7). The best-fit parameters are gathered in Table S1.

5. Full temperature-dependent (*ac*) magnetic characteristics of **1@Y** at H_{dc} = 1 kOe. (Figure S7)

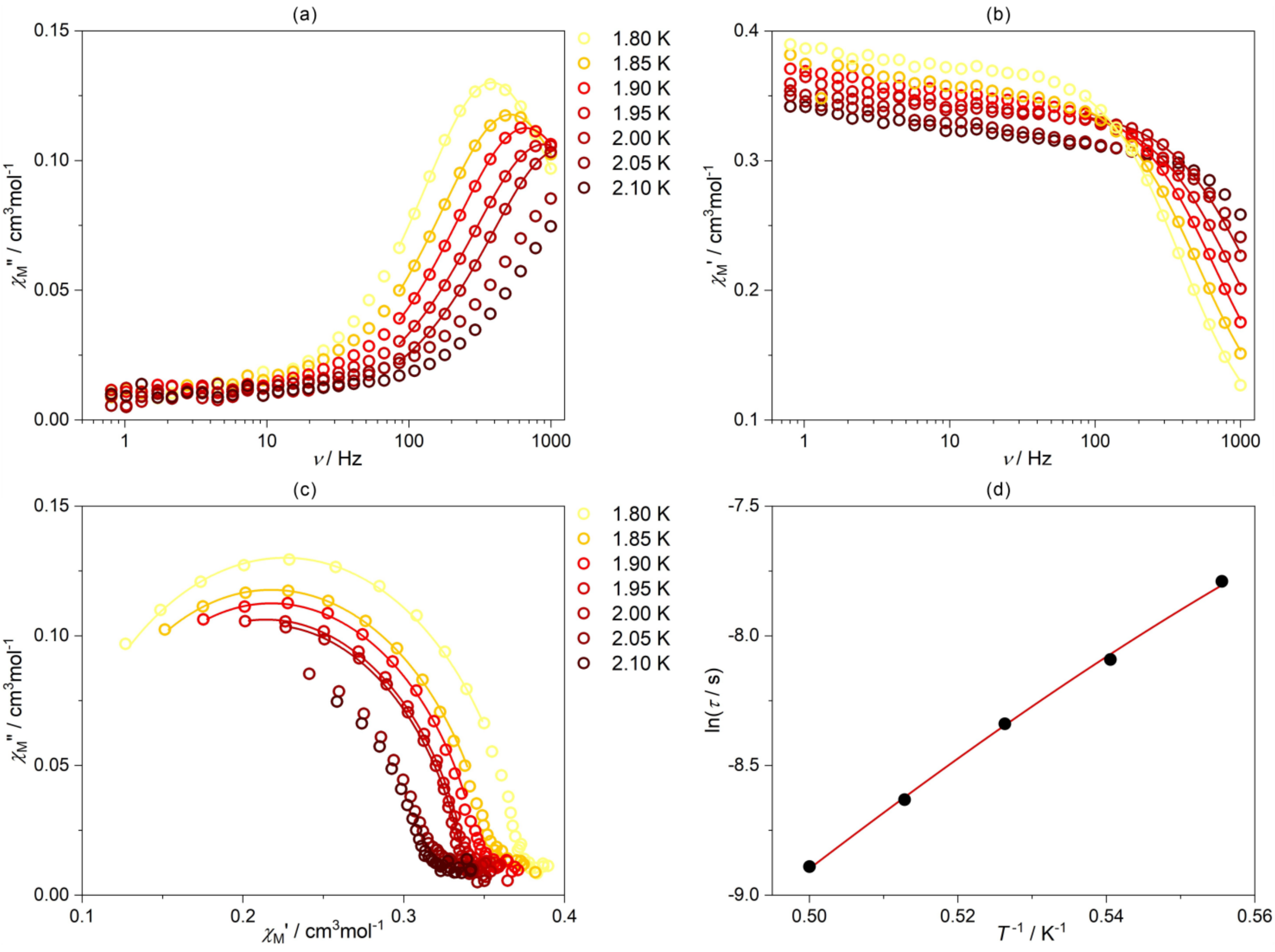

Figure S7. Full temperature-dependent alternate-current (*ac*) magnetic characteristics of **1@Y** at the optimal *dc* field of 1 kOe (the representative curves are shown in Figure 2): frequency dependences of the out-of-phase susceptibility, χ_M" (*a*) and the in-phase susceptibility, χ_M' (*b*) at the indicated temperatures, together with the related Argand plots (*c*), and the resulting temperature dependence of the relaxation time (*d*). The empty points in (*a*–*c*) represent the experimental data while the respective solid lines show the fitting according to the generalized Debye model. The black points in (*d*) represent the experimental data while the red solid line shows the best-fit curve related to the combined contributions from QTM, direct, and Orbach relaxation processes (the simultaneous fit together with the related field-dependence of the relaxation time, Figure S6). The alternative fittings for the temperature-dependence of the relaxation time is presented in Figure S8. The best-fit parameters are gathered in Table S1.

6. The comparison of the alternative fittings of the temperature dependence of magnetic relaxation time detected for **1@Y** at the optimal *dc* field of 1 kOe. (Figure S8)

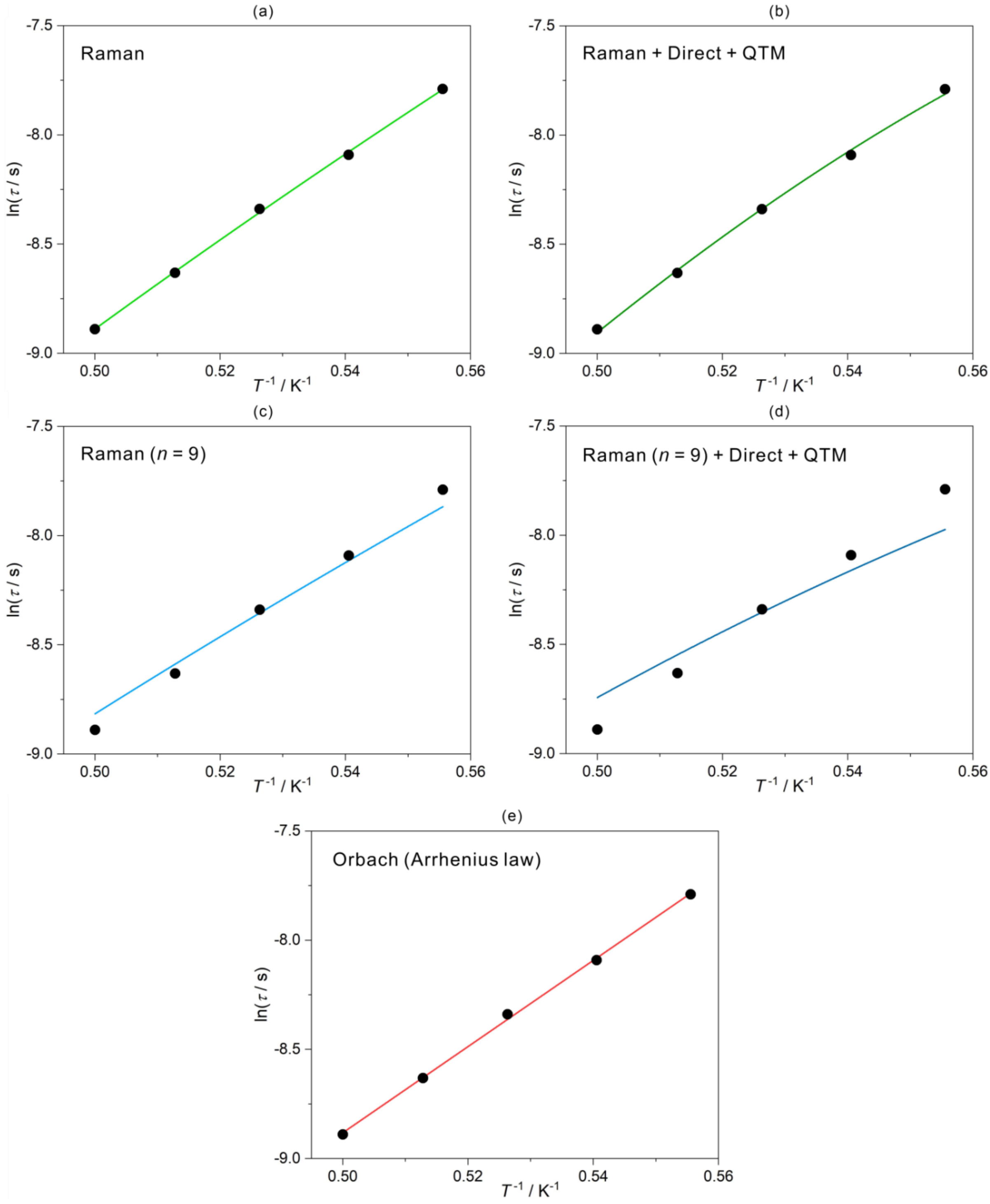

Figure S8. The comparison of the alternative fittings of the temperature dependence of magnetic relaxation time detected for **1@Y** at the optimal *dc* field of 1 kOe: the experimental points (black circles) and the best-fit curves (coloured lines) obtained taking into account only Raman relaxation (*a*), the combination of Raman, direct, and QTM processes (*b*), only Raman relaxation with the fixed power *n* of 9, as expected for the Kramers ions (*c*), the combination of direct, QTM, and Raman relaxation with the fixed power *n* of 9 (*d*), and only Orbach relaxation (Arrhenius law) (*e*). The best-fit parameters are gathered in Table S2.

7. Alternate-current (*ac*) magnetic properties of **2**, including *dc*-field-variable characteristics at 1.8 K, and temperature-variable characteristics at 1 kOe. (Figure S9)

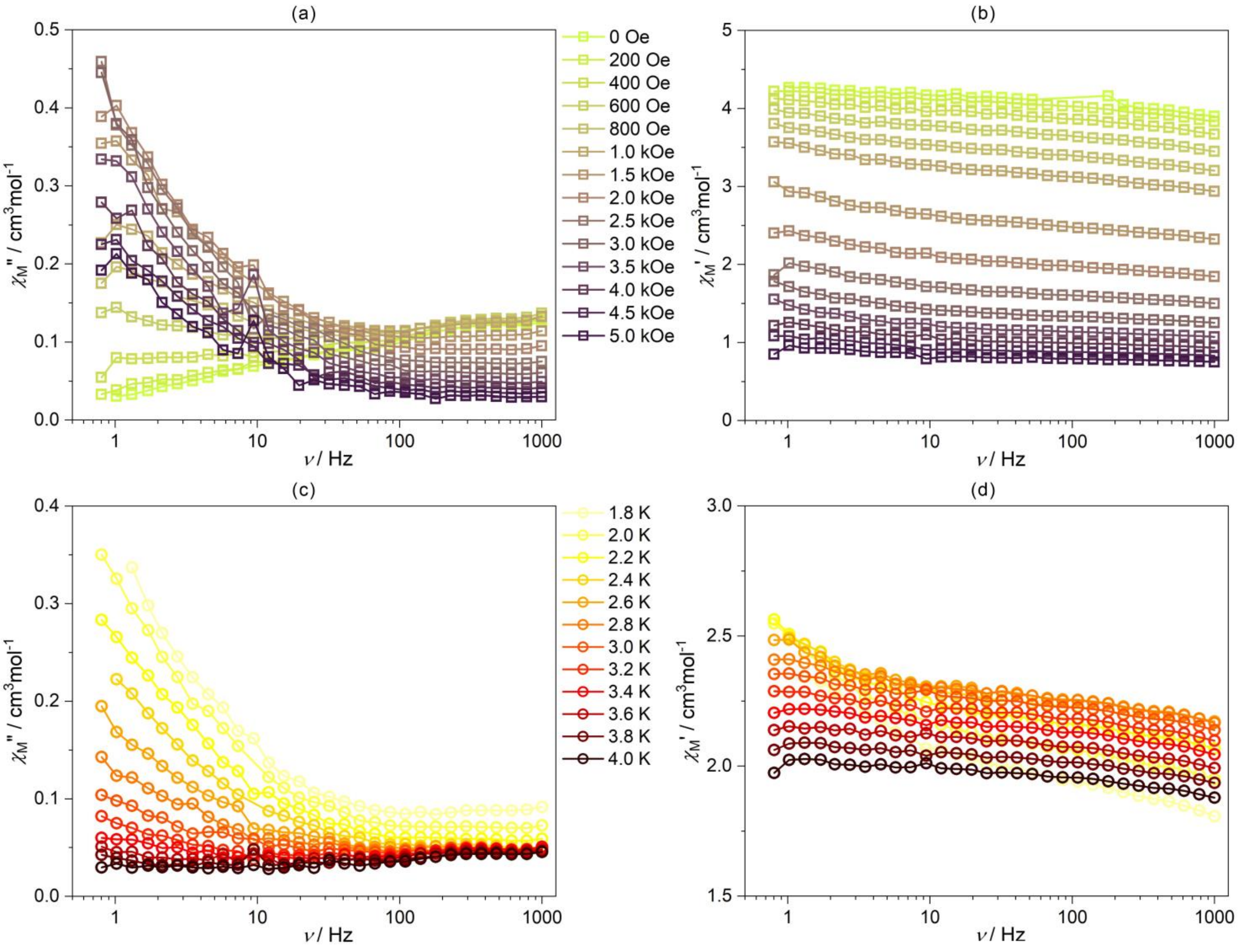

Figure S9. Alternate-current (*ac*) magnetic properties of **2**: *dc*-field-variable frequency dependences of the out-of-phase susceptibility, χ_M” (*a*) and the in-phase susceptibility, χ_M’ (*b*) collected in the 0–5 kOe range at T = 1.8 K, temperature-variable frequency dependences of the χ_M” (*c*) and χ_M’ (*d*) gathered in the 1.8–4.0 K range at H_{dc} = 1 kOe. The solid lines in (*a*–*d*) are only to guide the eye. The experimental curves were not fitted due to the lack of χ_M” maxima in the investigated frequency range.

8. Full alternate-current (*ac*) magnetic properties of **2@Y**, including *dc*-field-variable characteristics at 1.8 K, and temperature-variable characteristics at 1 kOe. (Figure S10)

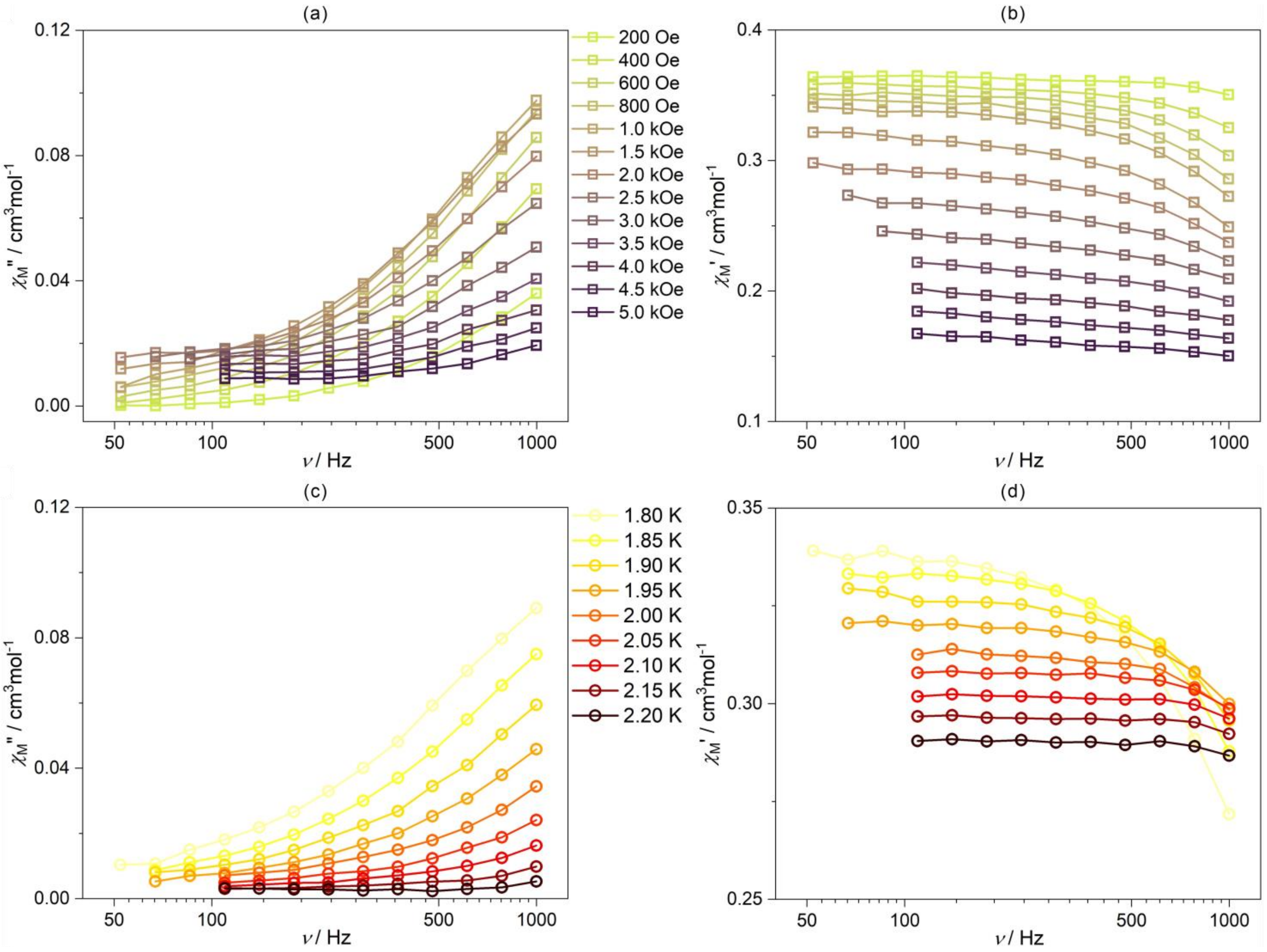

Figure S10. Full alternate-current (*ac*) magnetic properties of **2@Y**: *dc*-field-variable frequency dependences of the out-of-phase susceptibility, χ_M" (*a*) and the in-phase susceptibility, χ_M' (*b*) collected in the 0–5 kOe range at T = 1.8 K, temperature-variable frequency dependences of the χ_M" (*c*) and χ_M' (*d*) gathered in the 1.8–2.2 K range at H_{dc} = 1 kOe. The solid lines in (*a*–*d*) are only to guide the eye. The experimental curves were not fitted due to the lack of χ_M" maxima in the investigated frequency range, only the simplified approach utilizing the temperature dependence of ln(χ_M"/χ_M') was applied which is presented in Figure 2.

9. Best-fit parameters for the fittings of temperature- and/or *dc*-field-variable magnetic relaxation times for **1**, **2**, **1@Y**, and **2@Y**. (Table S1)

Table S1. Best-fit parameters for the fittings of temperature- and/or *dc*-field-variable magnetic relaxation times for **1**, **2**, **1@Y**, and **2@Y**.

compound	**1**		**2**	**1@Y**	**2@Y**
type of fitting	a simplified approach for the determination of the Arrhenius-type relaxation from the linear course of the ln(χ_M"/χ_M') = f(T^{-1}) plot[S1–S2]	the equation (1) (main text) with the only contribution from Orbach relaxation (Arrhenius-type relaxation)	any of the fitting procedures cannot be used (relaxation times are out of range)	simultaneous fit of the field-variable data at 1.8 K and *T*-variable data at 1 kOe following the equation (1) (main text)	a simplified approach for the determination of the Arrhenius-type relaxation from the linear course of the ln(χ_M"/χ_M') = f(T^{-1}) plot[S1–S2]
dc field / Oe	1000<br>(Figure S4)	2500<br>(Figure S5)	2000<br>(Figure S9)	1000<br>(Figures S6–S7)	1000<br>(Figure S10)
U_{eff} / K or $\Delta E/k_B$ / K	**U_{eff} = 2.29(13) K**<br>LT regime<br>**U_{eff} = 1.67(6) K**<br>HT regime<br>(average slope for the ln(χ_M"/χ_M') = f(T^{-1}) plots)	**$\Delta E/k_B$ = 7.23(13) K**<br>LT regime<br>**2.5(3) K**<br>HT regime	-	**$\Delta E/k_B$ = 24.6(5) K**	**U_{eff} = 20.1(3) K**<br>(average slope for the ln(χ_M"/χ_M') = f(T^{-1}) plots)
τ_0 / s	not determined by this method	**$2.44(13)\cdot10^{-3}$**<br>LT regime<br>**$1.20(9)\cdot10^{-2}$**<br>HT regime	-	**$7(2)\cdot10^{-10}$**	not determined by this method
A / $s^{-1}K^{-1}$ Oe^{-4}	-	-	-	$1.58(3)\cdot10^{-10}$	-
B_1 / s^{-1}	-	-	-	2619(136)	-
B_2 / Oe^{-2}	-	-	-	$4.1(6)\cdot10^{-6}$	-
B_{Raman} / $s^{-1}K^{-n}$	-	-	-	-	-
n	-	-	-	-	-

References:

S1. Ferrando-Soria, J.; Cangussu, D.; Eslava, M.; Journaux, Y.; Lescouëzec, R.; Julve, M.; Lloret, F.; Pasán, J.; Ruiz-Pérez, C.; Lhotel, E.; et al. Rational enantioselective design of chiral heterobimetallic single-chain magnets: Synthesis, crystal structures and magnetic properties of oxamato-bridged $M^{II}Cu^{II}$ chains (M=Mn, Co). *Chem.–A Eur. J.* **2011**, *17*, 12482–12494.

S2. Chorazy, S.; Nakabayashi, K.; Imoto, K.; Mlynarski, J.; Sieklucka, B.; Ohkoshi, S. I. Conjunction of chirality and slow magnetic relaxation in the supramolecular network constructed of crossed cyano-bridged Co^{II}-W^{V} molecular chains. *J. Am. Chem. Soc.* **2012**, *134*, 16151–16154.

10. Best-fit parameters for various possible fittings of the field- and temperature-dependent magnetic relaxation times in **1@Y**. (Table S2)

Table S2. Best-fit parameters for various possible fittings of the field- and temperature-dependent magnetic relaxation times in **1@Y** (Figure S8). Note that the parameters for the final fitting using Orbach, Direct, and QTM contributions were obtained within the simultaneous fit of both field- and temperature-dependences of relaxation time while for other alternative fittings, the parameters of Direct and QTM processes (if applied) were taken from that simultaneous fit, and used as the fixed values for the investigation of other possible relaxation routes.

types of relaxation processes taken into account for the global fitting (following the equation (7), main text)	Orbach + Direct + QTM (final fitting, Figure 2)	only Raman (Figure S8a)	Raman + Direct + QTM (Figure S8b)	only Raman with the fixed power *n* (Figure S8c)	Raman with the fixed power *n* + Direct + QTM (Figure S8d)	only Orbach (Figures 2 and S8e)
$\Delta E/k_B$ / K	24.6(5)	-	-	-	-	19.7(4)
τ_0 / s	$7(2)\cdot10^{-10}$	-	-	-	-	$7.2(13)\cdot10^{-9}$
A / $s^{-1}K^{-1}Oe^{-4}$	$1.58(3)\cdot10^{-10}$	-	$1.58(3)\cdot10^{-10}$	-	$1.58(3)\cdot10^{-10}$	-
B_1 / s^{-1}	2619(136)	-	2619(136)	-	2619(136)	-
B_2 / Oe^{-2}	$4.1(6)\cdot10^{-6}$	-	$4.1(6)\cdot10^{-6}$	-	$4.1(6)\cdot10^{-6}$	-
B_{Raman} / $s^{-1}K^{-n}$	-	5.3(5)	0.84(19)	13.2(4)	10.6(8)	-
n	-	10.41(15)	12.9(3)	9 (fixed)	9 (fixed)	-

11. Details of the *ab initio* calculations for Dy^{III} complexes in the crystal structures of **1**–**4** (Tables S3–S9, Figure S11).

Computational details

The *ab initio* calculations of CASSCF/RASSI/SINGLE_ANISO[S3] type were carried out for **1**, **2**, **3**, and **4**, using the OpenMolcas quantum chemistry software package[S4]. They were performed on the experimental geometries taken from powder X-ray diffraction analysis without optimization. Molecular clusters consisting of Dy^{3+} central ion surrounded with BH_4^- units were considered. The fragments of the crystal structures employed for analyses are presented in Figure S11. Three models with different basis sets were used: **S** - small with VDZP basis function quality, **L** – large with VTZP basis, and **V** – very large employing VQZP functions. Tables S3 and S8 contain contractions and labels of the basis sets for all the atoms. Scalar relativistic effects were taken into account by employing two-component second-order Douglas-Kroll-Hess (DKH2) Hamiltonian together with relativistic Atomic Natural Orbital basis sets - ANO-RCC type.[S5–S6] In order to save disk space, the Cholesky decomposition of ERI-s (electron repulsion integrals) was used with the $1.0 \cdot 10^{-8}$ threshold. In the first step of the employed procedure, a State Average Multi-Configurational Self-Consistent Field (SA-CASSCF) calculation for 21 sextets, 224 quartets, and 490 doublets rising from different possible electron distributions for $4f^9$ configuration was performed. The active space was composed out of 7 f-orbitals with 9 active valence electrons – CAS(9in7). In the next step, all sextets, 128 quartets, and 130 doublets optimized as spin-free states in the CASSCF step were mixed by the spin-orbit coupling within RASSI (Restricted Active Space State Interaction Program)[S7] using mean-field spin-orbit (SO) integrals (AMFI)[S8] resulting in 898 spin-orbit states In the final step, a SINGLE_ANISO[S9] module was used to decompose spin-orbit states into states with a definite projection of the total momentum on the located quantization axis and to extract three components of the pseudo-*g*-tensor for eight ground Kramers doublets. The obtained energy splitting of the J = 15/2 manifold, together with the g_x, g_y, g_z components of the pseudo-g-tensors within the basis of each doublet ($\tilde{S} = 1/2$) and decomposition of the ground state into states with definite angular momentum on the quantization axis are presented in Tables S4–S7 and S9.

References:

S3. Chibotaru, L. F.; Ungur, L. Ab initio calculation of anisotropic magnetic properties of complexes. I. Unique definition of pseudospin Hamiltonians and their derivation. *J. Chem. Phys.* **2012**, *137*, 064112.

S4. Galvam I. F.; Vacher, M.; Alavi, A.; Angeli, C.; Aquilante, F.; Autschbach, J.; Bao, J. J.; Bokarev, S. I.; Bogdanov, N. A.; Carlson, R. K.;Chibotaru, L. F.; Creutzberg, J.; Dattani, N.; Delcey, M. G.; Dong, S. S.; Dreuw, A.; Freitag, L.; Frutos, L. M.; Gagliardi, L.; Gendron, F.; Giussani, A.; Gonzalez, L.; Grell, G.; Guo, M.; Hoyer, C. E.; Johansson, M.; Keller, S.; Knecht, S.; Kovacevic, G.; Kallman, E.; Manni, G. L.; Lundberg, M.; Ma, Y.; Mai, S.; Malhado, J. P.; Malmqvist, P. Å.; Marquetand, P.; Mewes, S. A.; Norell, J.; Olivucci, M.; Oppel, M.; Phung, Q. M.; Perloot, K.; Plasser, F.; Reiher, M.; Sand, A. M.; Schapiro, I.; Sharma, P.; Stein, C. J.; Sorensen, L. K.; Truhlar, D. G.; Ugandi, M.; Ungur, L.; Valentini, A.; Vancoillie, S.; Veryazov, V.; Weser, O.; Wesołowski, T. A.; Widmark, P.-O.; Wouters, S.; Zech, A.; Zobel, J. P.; Lindh, R. OpenMolcas: From Source Code to Insight. *J. Chem. Theory Comput.* **2019**, *15*, 5925–5964.

S5. Roos, B. O.; Lindh, R.; Malmqvist, P. Å.; Veryazov, V.; Widmark, P. O. Main group atoms and dimers studied with a new relativistic ANO basis set. *J. Phys. Chem. A* **2004**, *108*, 2851–2858.

S6. Roos, B. O.; Lindh, R.; Malmqvist, P. Å.; Veryazov, V.; Widmark, P. O.; Borin, A. C. New Relativistic Atomic Natural Orbital Basis Sets for Lanthanide Atoms with Applications to the Ce Diatom and LuF_3. *J. Phys. Chem. A* **2008**, *112*, 11431–11435.

S7. Malmqvist, P. Å.; Roos, B. O.; Schimmelpfennig, B. The Restricted Active Space (RAS) State Interaction Approach with Spin-Orbit Coupling. *Chem. Phys. Lett.* **2002**, *357*, 230−240.

S8. Heß, B. A.; Marian, C. M.; Wahlgren, U.; Gropen, O. A Mean-Field Spin-Orbit Method Applicable to Correlated Wavefunctions. *Chem. Phys. Lett.* **1996**, *251*, 365−371.

S9. Ungur, L.; Chibotaru, L. F. Ab Initio Crystal Field for Lanthanides. *Chem. Eur. J.* **2017**, *23*, 3708–3718.

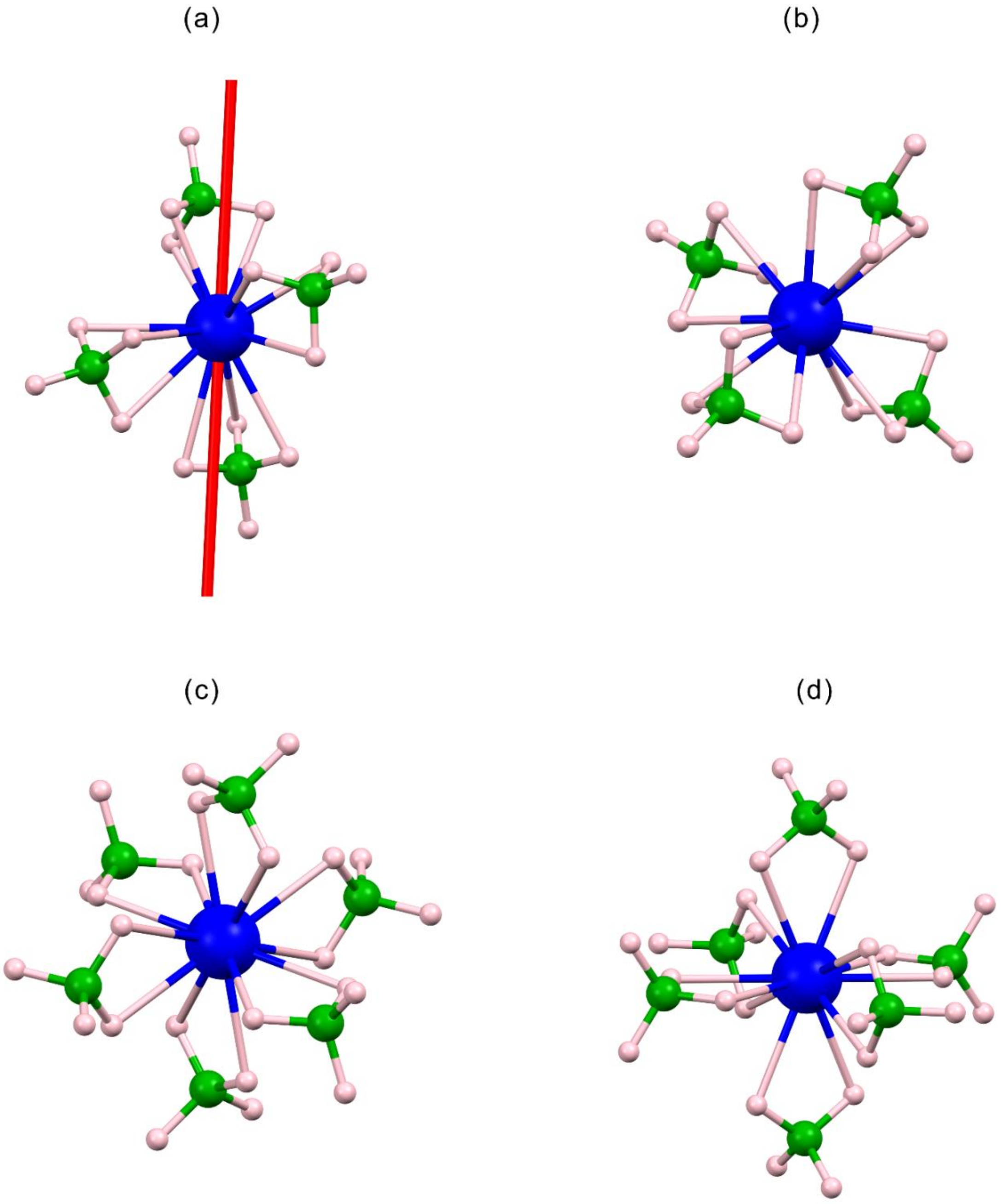

Figure S11. The structural fragments of **1** (*a*), **2** (*b*), **3** (*c*), and **4** (*d*), used for the *ab initio* calculations. The color code: Dy – dark blue, B – green, H – light pink. The red line in (*a*) represents the alignment of the magnetic easy axis of the ground state obtained within the large basis (Table S4). The other compounds do not show an easy-axis type of magnetic anisotropy, thus the magnetic axes were not shown.

Table S3. Description and contractions of the basis sets (two models: **S** - small, **L** - large) employed in the *ab initio* calculations of the Dy^{III} crystal field in **1**, **2**, **3,** and **4**.

**Basis set S**	**Basis set L**
Dy.ANO-RCC-VDZP 7S6P4D2F1G	Dy.ANO-RCC-VTZP 8S7P5D3F2G1H
B.ANO-RCC-VDZP 3S2P1D	B.ANO-RCC-VTZP 4S3P2D1F
H.ANO-RCC-VDZP 2S1P	H.ANO-RCC-VTZP 3S2P1D

Table S4. Summary of the energy splitting of the $^6H_{15/2}$ multiplet of Dy^{III} in **1** using models **S** and **L** with pseudo-*g*-tensors of each Kramers doublet and the composition in the $|m_J\rangle$ basis of the ground state.

**1**							
**S**				**L**			
**Energy and pseudo-*g*-tensor components (g_x, g_y, g_z) of 8 ground Kramers doublets**							
Energy / cm^{-1}	Pseudo-*g*-tensor components			Energy / cm^{-1}	Pseudo-*g*-tensor components		
	g_x	g_y	g_z		g_x	g_y	g_z
0.000	0.0245	0.0416	19.5437	**0.000**	**0.0261**	**0.0444**	**19.5407**
100.227	0.2677	0.3458	16.7932	**104.068**	**0.2837**	**0.3672**	**16.7808**
189.797	1.7234	2.2228	13.4232	196.866	1.8245	2.3379	13.3323
245.994	5.1267	6.0260	7.8444	255.178	5.2157	6.1437	7.5929
295.448	0.3381	3.9511	9.4623	307.073	0.4919	3.7070	9.5227
352.886	0.9072	2.5634	11.7734	367.015	0.9475	2.4964	11.9235
403.622	1.0169	1.9815	15.5458	421.274	0.9855	1.8860	15.5950
513.313	0.0394	0.0548	19.4836	538.969	0.0373	0.0559	19.4869
**Composition of the ground Kramers doublet in the $|m_J\rangle$ basis on the quantization axis within *J* = 15/2 manifold**							
63.3% $|+15/2\rangle$<br>31.0% $|-15/2\rangle$<br>3.2% $|-11/2\rangle$<br>1.5% $|+11/2\rangle$<br>0.3% $|-9/2\rangle$<br>0.2% $|+9/2\rangle$				94.3% $|+15/2\rangle$<br>4.8% $|-11/2\rangle$<br>0.4% $|-9/2\rangle$<br>0.1% $|-5/2\rangle$			

Table S5. Summary of the energy splitting of the $^6H_{15/2}$ multiplet of Dy^{III} in **2** using models **S** and **L** with pseudo-*g*-tensors of each Kramers doublet and the composition in the $|m_J\rangle$ basis of the ground state.

**2**							
**S**				**L**			
**Energy and pseudo-*g*-tensor components (g_x, g_y, g_z) of 8 ground Kramers doublets**							
Energy / cm^{-1}	Pseudo-*g*-tensor components			Energy / cm^{-1}	Pseudo-*g*-tensor components		
	g_x	g_y	g_z		g_x	g_y	g_z
0.000	9.8939	9.8934	2.3747	**0.000**	**9.8998**	**9.8988**	**2.3685**
26.687	2.9869	2.9873	7.1212	**28.003**	**3.0981**	**3.0990**	**7.0675**
68.829	7.1646	7.1196	1.3069	73.100	6.9973	6.9885	1.3234
69.085	9.4956	9.4507	0.2996	73.616	9.4758	9.4670	0.2957
103.769	3.2495	3.2495	8.4637	109.953	3.3839	3.3840	8.3782
175.129	0.8703	0.8704	12.5975	184.882	0.8870	0.8871	12.5857
205.044	0.0674	0.0675	18.5157	215.739	0.0728	0.0729	18.4991
237.151	1.2555	1.2555	15.7716	250.134	1.2770	1.2770	15.7591
**Composition of the ground Kramers doublet in the $|m_J\rangle$ basis on the quantization axis within *J* = 15/2 manifold**							
43.9% $|+5/2\rangle$<br>31.2% $|-3/2\rangle$<br>10.1% $|-5/2\rangle$<br>7.1% $|+3/2\rangle$<br>3.7% $|-13/2\rangle$<br>2.6% $|-11/2\rangle$<br>0.8% $|+13/2\rangle$<br>0.6% $|-11/2\rangle$				49.6% $|+5/2\rangle$<br>35.3% $|-3/2\rangle$<br>4.3% $|-5/2\rangle$<br>4.2% $|+13/2\rangle$<br>3.1% $|+3/2\rangle$<br>2.9% $|-11/2\rangle$<br>0.4% $|-13/2\rangle$			

Table S6. Summary of the energy splitting of the $^{6}H_{15/2}$ multiplet of Dy^{III} in **3** using models **S** and **L** with pseudo-*g*-tensors of each Kramers doublet and the composition in the $|m_J\rangle$ basis of the ground state.

**3**							
**S**				**L**			
**Energy and pseudo-*g*-tensor components (g_x, g_y, g_z) of 8 ground Kramers doublets**							
Energy / cm^{-1}	Pseudo-*g*-tensor components			Energy / cm^{-1}	Pseudo-*g*-tensor components		
	g_x	g_y	g_z		g_x	g_y	g_z
0.000	1.1452	5.0206	12.9985	**0.000**	**10.9028**	**8.7513**	**0.4911**
0.001	11.6305	7.7556	0.2217	**0.000**	**1.8726**	**4.0240**	**13.2664**
32.571	5.9390	5.9385	5.9380	33.547	5.9615	5.9615	5.9614
128.812	6.8186	6.8186	6.8183	133.864	6.8409	6.8410	6.8412
140.305	1.2156	1.8361	9.8746	145.119	0.5618	4.4074	9.0880
140.305	7.4020	6.7814	1.2574	145.120	0.4657	4.2145	9.1843
230.069	4.9457	5.2810	10.8290	237.241	0.3814	2.1911	12.8260
230.069	2.1100	3.5148	12.3617	237.242	5.4812	6.9628	9.5358
**Composition of the ground Kramers doublet in the $|m_J\rangle$ basis on the quantization axis within *J* = 15/2 manifold**							
49.5% $|+15/2\rangle$<br>19.5% $|+11/2\rangle$<br>16.2% $|-1/2\rangle$<br>7.7% $|+7/2\rangle$<br>2.3% $|-5/2\rangle$<br>1.9% $|-9/2\rangle$				37.1% $|+3/2\rangle$<br>24.7% $|-5/2\rangle$<br>10.4% $|-3/2\rangle$<br>6.2% $|-9/2\rangle$<br>5.1% $|-1/2\rangle$<br>3.9% $|+7/2\rangle$<br>1.7% $|+9/2\rangle$<br>1.0% $|-7/2\rangle$<br>0.9% $|+15/2\rangle$			

Table S7. Summary of the energy splitting of the $^{6}H_{15/2}$ multiplet of Dy^{III} in **4** using models **S** and **L** with pseudo-*g*-tensors of each Kramers doublet and the composition in the $|m_J\rangle$ basis of the ground state.

**4**							
**S**				**L**			
**Energy and pseudo-*g*-tensor components (g_x, g_y, g_z) of 8 ground Kramers doublets**							
Energy / cm^{-1}	Pseudo-*g*-tensor components			Energy / cm^{-1}	Pseudo-*g*-tensor components		
	g_x	g_y	g_z		g_x	g_y	g_z
0.000	8.5903	8.5902	4.8047	**0.000**	**8.5766**	**8.5763**	**4.8434**
16.296	0.0000	0.0000	11.4662	**16.577**	**0.0000**	**0.0000**	**11.5167**
48.764	6.6709	6.6709	3.7166	49.852	6.7004	6.7003	3.7944
98.635	8.2405	8.2400	0.1511	102.480	8.2414	8.2404	0.1167
103.346	0.0002	0.0002	2.8752	107.036	0.0004	0.0005	2.9424
141.505	4.1919	4.1919	6.7722	146.171	4.1373	4.1373	6.7791
179.446	3.7332	3.7332	12.1512	185.317	3.6349	3.6349	12.2314
203.473	0.0000	0.0000	16.6553	210.163	0.0000	0.0000	16.6838
**Composition of the ground Kramers doublet in the $|m_J\rangle$ basis on the quantization axis within *J* = 15/2 manifold**							
44.5% $|+7/2\rangle$<br>21.8% $|-5/2\rangle$<br>16.9% $|+11/2\rangle$<br>7.8% $|-11/2\rangle$<br>3.0% $|-7/2\rangle$<br>2.7% $|+1/2\rangle$<br>1.5% $|+5/2\rangle$<br>1.1% $|-13/2\rangle$				46.9% $|+7/2\rangle$<br>22.7% $|-5/2\rangle$<br>17.6% $|+13/2\rangle$<br>8.1% $|-11/2\rangle$<br>2.6% $|+1/2\rangle$<br>1.0% $|-7/2\rangle$<br>0.4% $|-13/2\rangle$			

Table S8. Description and contractions of the basis set **V** employed in the *ab initio* calculations of the Dy^{III} crystal field in **1** and **2**.

**Basis set V**
Dy.ANO-RCC-VQZP 9S8P6D4F3G2H
B.ANO-RCC-VQZP 5S4P3D2F1G
H.ANO-RCC-VQZP 4S3P2D1F

Table S9. Summary of the energy splitting of the $^6H_{15/2}$ multiplet of Dy^{III} in **1** and **2** using model **V** with pseudo-*g*-tensors of each Kramers doublet and the composition in the $|m_J\rangle$ basis of the ground state.

**V basis**							
**1**				**2**			
**Energy and pseudo-*g*-tensor components (g_x, g_y, g_z) of 8 ground Kramers doublets**							
Energy / cm^{-1}	Pseudo-*g*-tensor components			Energy / cm^{-1}	Pseudo-*g*-tensor components		
	g_x	g_y	g_z		g_x	g_y	g_z
0.000	0.0244	0.0413	19.5524	0.000	9.9150	9.9148	2.3283
105.571	0.2688	0.3478	16.7881	28.235	3.2710	3.2711	6.8771
199.898	1.7152	2.1884	13.4082	73.515	6.9082	6.9043	1.0221
260.080	5.2421	6.0332	7.6962	74.141	9.4987	9.4948	0.2302
312.750	0.2524	3.8454	9.4989	110.794	3.3551	3.3552	8.5162
373.788	0.9040	2.4045	11.9446	186.053	0.8672	0.8672	12.6443
429.321	0.9515	1.8244	15.6282	217.590	0.0723	0.0723	18.5262
548.392	0.0376	0.0547	19.4923	251.642	1.2469	1.2469	15.7925
**Composition of the ground Kramers doublet in the $|m_J\rangle$ basis on the quantization axis within *J* = 15/2 manifold**							
93.4% $|+15/2\rangle$<br>4.5% $|+11/2\rangle$<br>1.1% $|-15/2\rangle$<br>0.5% $|+9/2\rangle$				52.6% $|+5/2\rangle$<br>37.9% $|-3/2\rangle$<br>4.3% $|+13/2\rangle$<br>3.0% $|-11/2\rangle$<br>1.2% $|-5/2\rangle$<br>0.8% $|+3/2\rangle$			

12. Solid-state low-temperature (77 K) photoluminescent properties of **1** and **2**. (Figure S12)

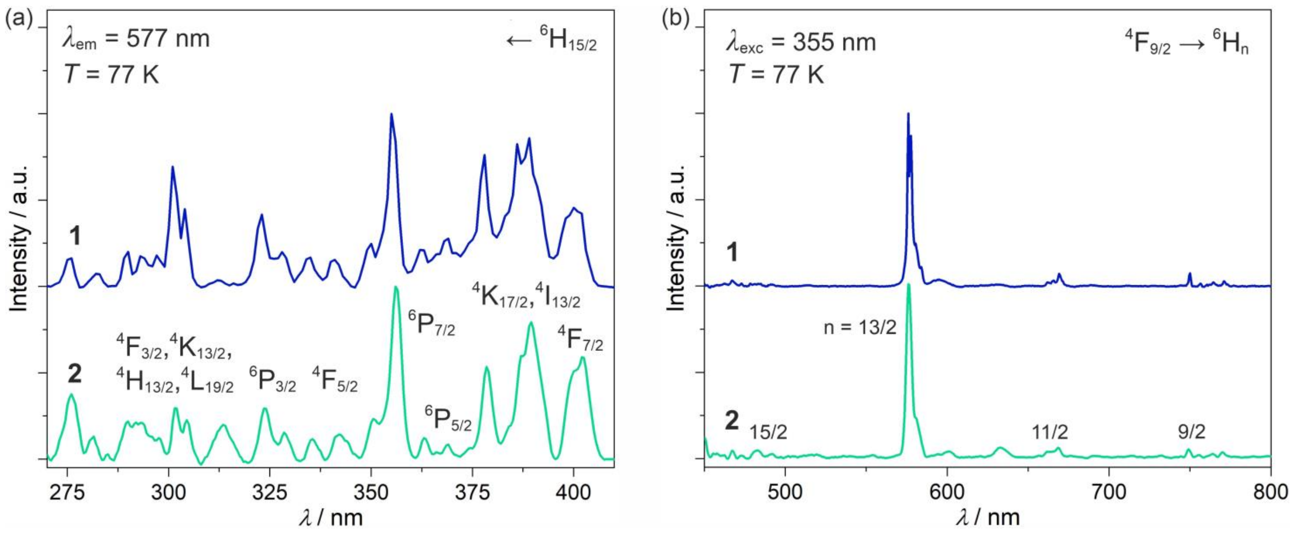

Figure S12. Solid-state low-temperature (77 K) photoluminescent properties of **1** and **2**: excitation spectra for the monitored emission at 577 nm with the indicated f-f electronic transitions (*a*), and the respective emission spectra for the excitation wavelength of 355 nm with the indicated emissive f-f electronic transitions (*b*).

13. Rietveld refinements for **1@Y**, **2@Y**, **1**, and **2**, (Figures S13–S16) and crystal structures of α-$Dy(BH_4)_3$ and β-$Dy(BH_4)_3$ (Figure S17)

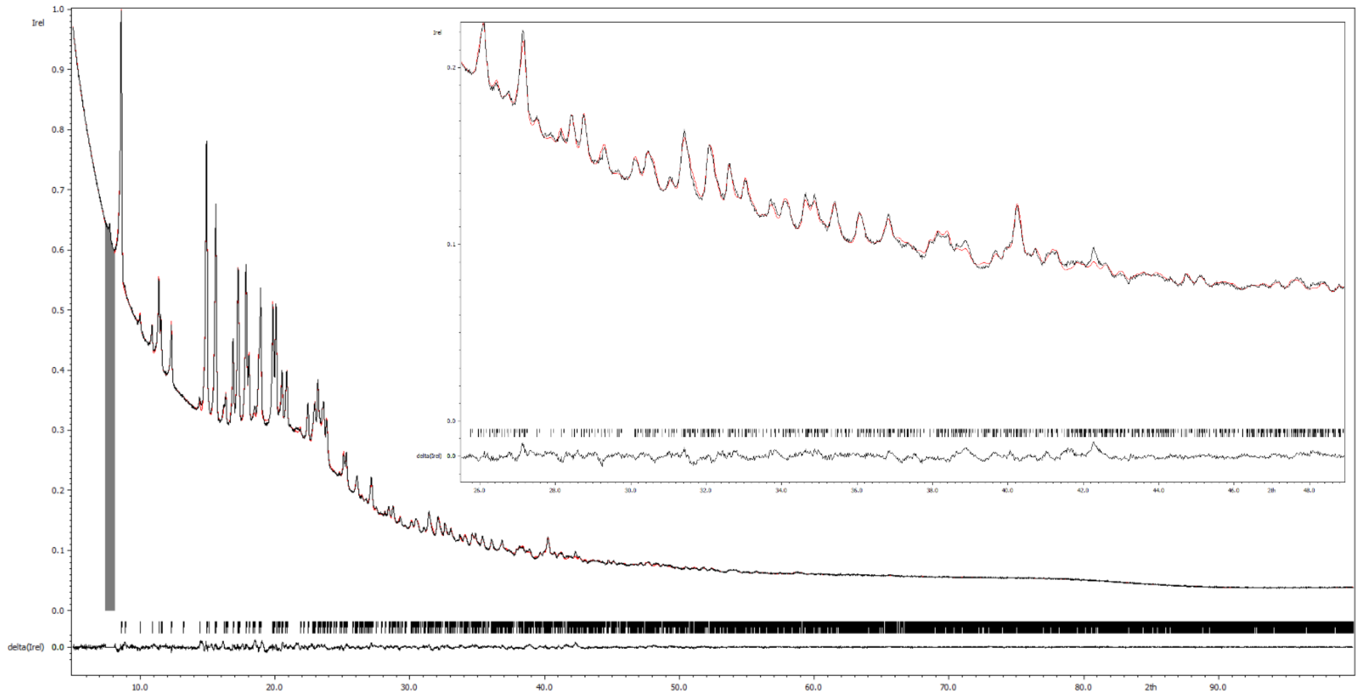

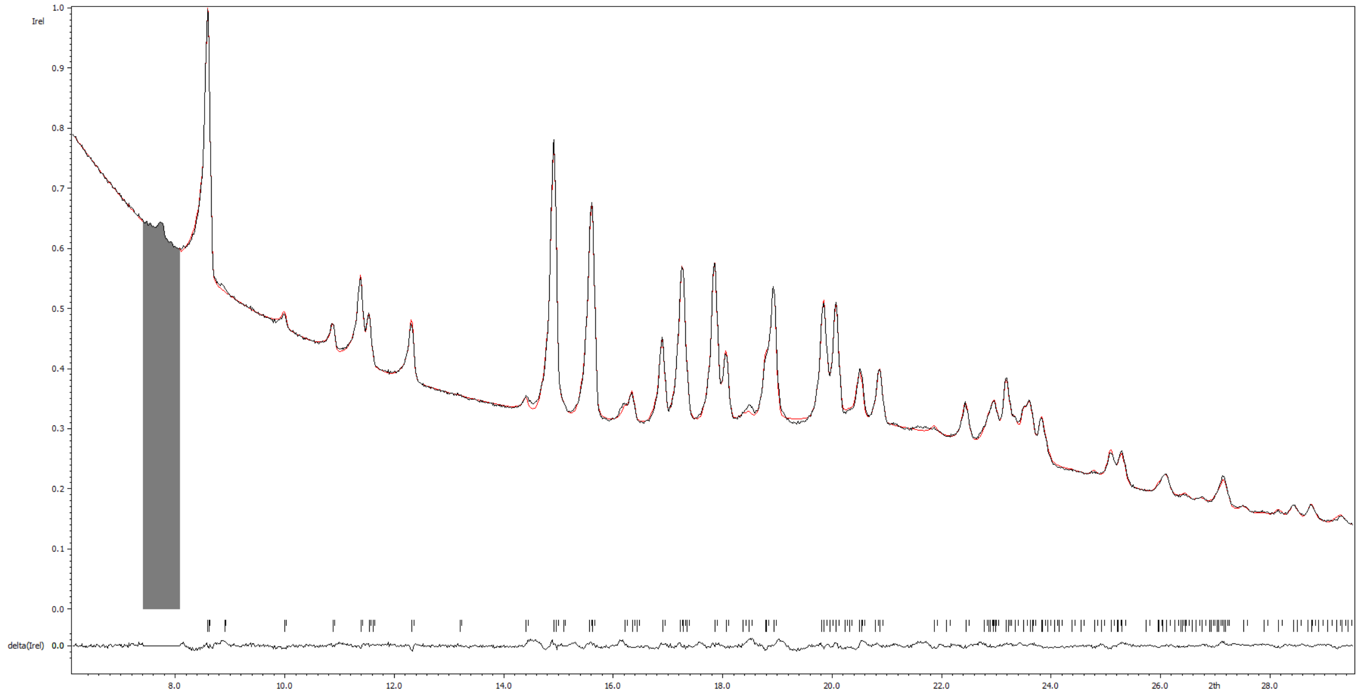

Figure S13. Rietveld refinement for recrystallized sample **1@Y** containing only [n-Bu_4N][$Y_{0.9}Dy_{0.1}(BH_4)_4$]. Experimental data are represented by a black curve, calculated profile by a red curve. The positions of the Bragg reflections are marked and the difference curve (between the experimental and calculated profiles) are plotted at the bottom of each figure. Inset: the low angle region. Bottom figure: the high angle region. Grey color indicates the regions, containing signals from impurities, excluded from the analysis.

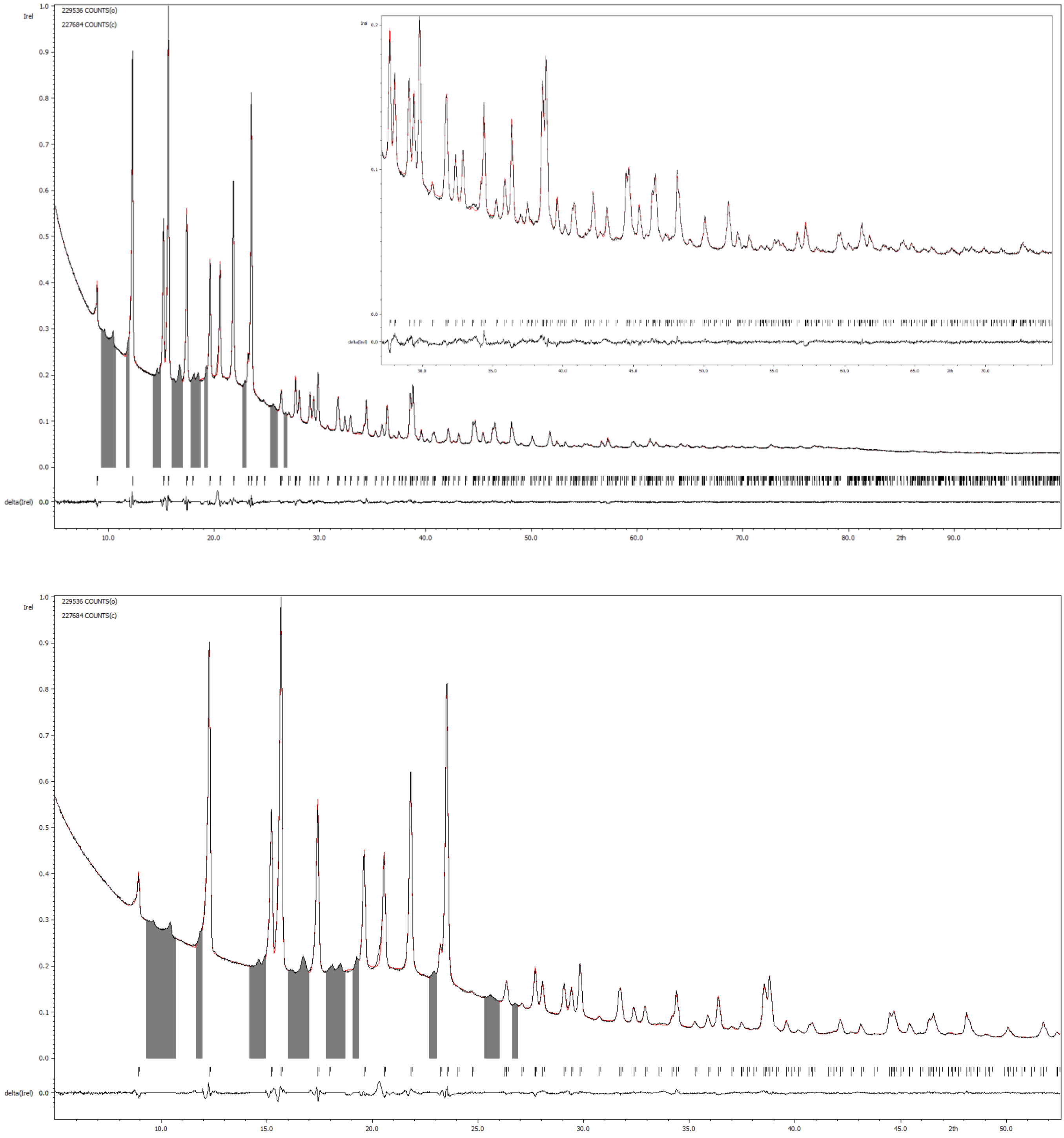

Figure S14. Rietveld refinement for recrystallized sample **2@Y** containing only $[Ph_4P][Y_{0.9}Dy_{0.1}(BH_4)_4]$. Experimental data are represented by a black curve, calculated profile by a red curve. The positions of the Bragg reflections are marked and the difference curve (between the experimental and calculated profiles) are plotted at the bottom of each figure. Inset: the low angle region. Bottom figure: the high angle region. Grey color indicates the regions, containing signals from impurities, excluded from the analysis.

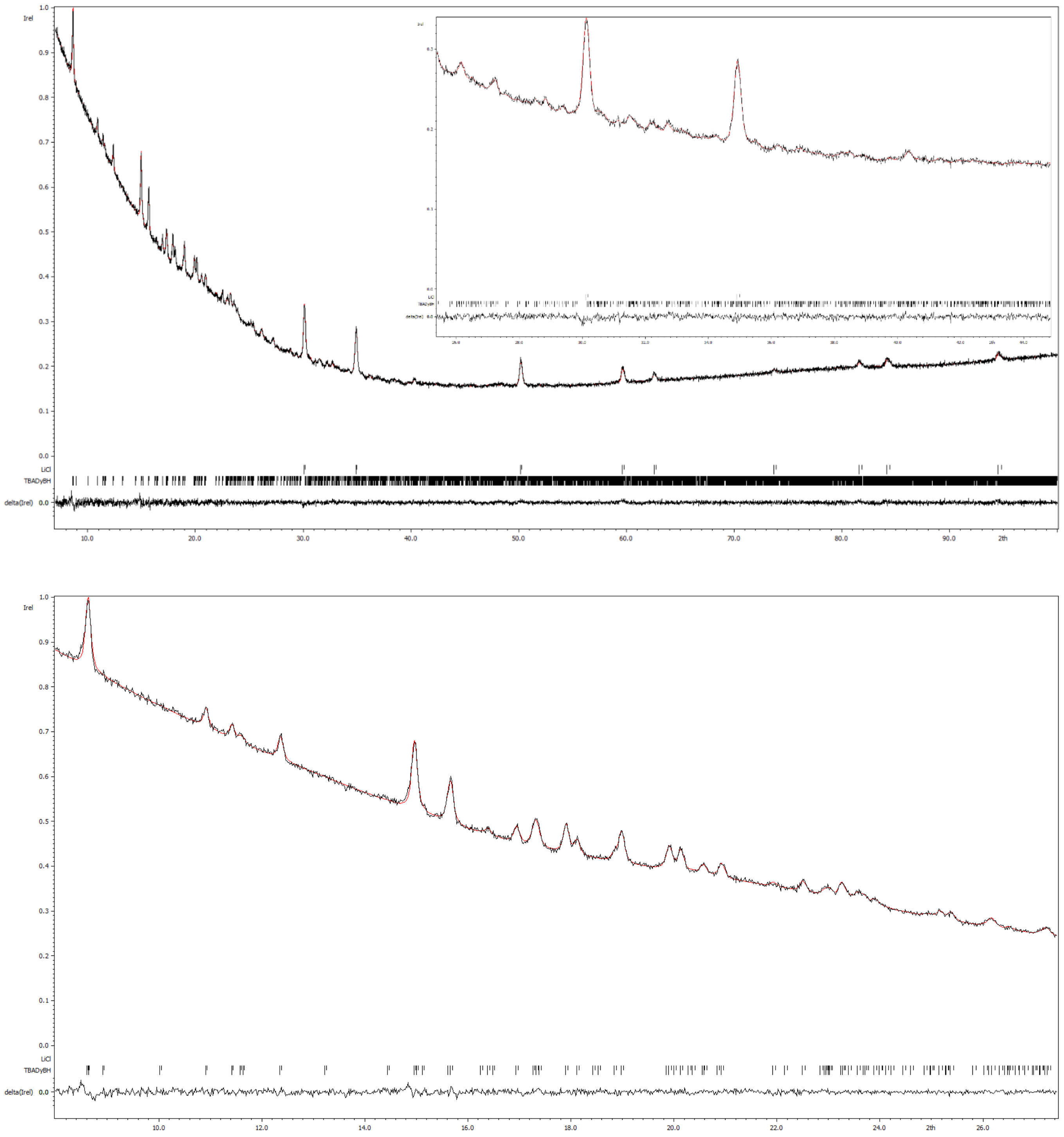

Figure S15. Rietveld refinement for as-milled sample **1** containing [n-Bu_4N][$Dy(BH_4)_4$] and LiCl. Experimental data are represented by a black curve, calculated profile by a red curve. The positions of the Bragg reflections are marked and the difference curve (between the experimental and calculated profiles) are plotted at the bottom of each figure. Inset: the low angle region. Bottom figure: the high angle region.

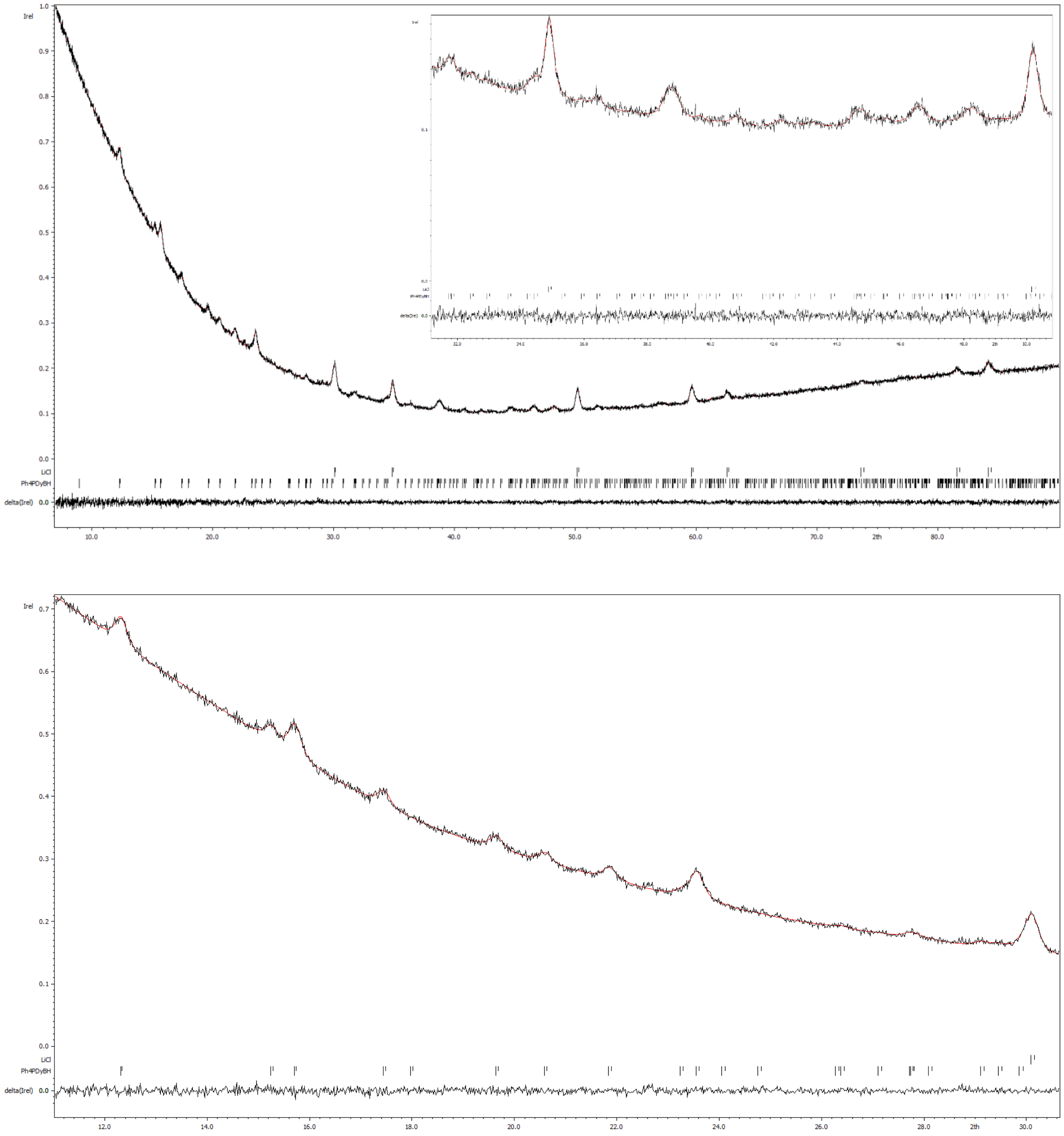

Figure S16. Rietveld refinement for as-milled sample **2** containing $[Ph_4P][Dy(BH_4)_4]$ and LiCl. Experimental data are represented by a black curve, calculated profile by a red curve. The positions of the Bragg reflections are marked and the difference curve (between the experimental and calculated profiles) are plotted at the bottom of each figure. Inset: the low angle region. Bottom figure: the high angle region.

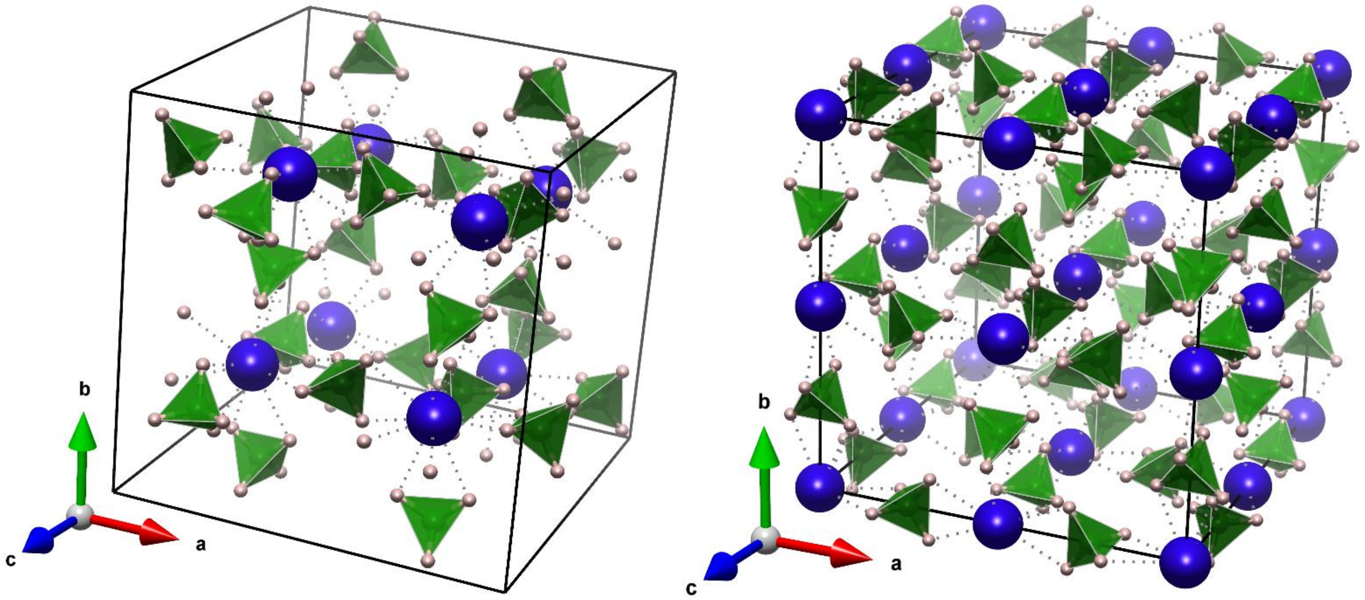

Figure S17. Crystal structures of α-$Dy(BH_4)_3$ (left) and β-$Dy(BH_4)_3$ (right). Dy: blue color, B and BH_4 polyhedral: green, H: pink.[S10]

Reference:

S10. Wegner, W.; Jaroń, T.; Grochala, W. Preparation of a series of lanthanide borohydrides and their thermal decomposition to refractory lanthanide borides. *J. Alloys Compd.* **2018**, *744*, 57–63.
